# A Deep Look Into COVID-19 Severity Through Dynamic Changes in Blood Cytokine Levels

**DOI:** 10.3389/fimmu.2021.771609

**Published:** 2021-11-09

**Authors:** Denis A. Kleymenov, Evgeniia N. Bykonia, Liubov I. Popova, Elena P. Mazunina, Vladimir A. Gushchin, Liudmila V. Kolobukhina, Olga A. Burgasova, Irina S. Kruzhkova, Nadezhda A. Kuznetsova, Elena V. Shidlovskaya, Elizaveta V. Divisenko, Andrei A. Pochtovyi, Valeria V. Bacalin, Svetlana V. Smetanina, Artem P. Tkachuk, Denis Y. Logunov, Alexander L. Gintsburg

**Affiliations:** ^1^ Federal State Budget Institution “National Research Centre for Epidemiology and Microbiology Named After Honorary Academician N. F. Gamaleya” of the Ministry of Health of the Russian Federation, Moscow, Russia; ^2^ Department of Virology, Biological Faculty, Lomonosov Moscow State University, Moscow, Russia; ^3^ Moscow Healthcare Department, Moscow, Russia; ^4^ Department of Infectious Diseases, Peoples’ Friendship University of Russia (RUDN University), Moscow, Russia; ^5^ Department of Infectiology and Virology, Federal State Autonomous Educational Institution of Higher Education I. M. Sechenov, First Moscow State Medical University of the Ministry of Health of the Russian Federation (Sechenov University), Moscow, Russia

**Keywords:** SARS-CoV-2, COVID-19, cytokines, severity predictors, hyperinflammation

## Abstract

An excessive inflammatory response to SARS-CoV-2 is thought to be a major cause of disease severity and mortality in patients with COVID-19. Longitudinal analysis of cytokine release can expand our understanding of the initial stages of disease development and help to identify early markers serving as predictors of disease severity. In this study, we performed a comprehensive analysis of 46 cytokines (including chemokines and growth factors) in the peripheral blood of a large cohort of COVID-19 patients (n=444). The patients were classified into five severity groups. Longitudinal analysis of all patients revealed two groups of cytokines, characterizing the “early” and “late” stages of the disease course and the switch between type 1 and type 2 immunity. We found significantly increased levels of cytokines associated with different severities of COVID-19, and levels of some cytokines were significantly higher during the first three days from symptom onset (DfSO) in patients who eventually required intensive care unit (ICU) therapy. Additionally, we identified nine cytokines, TNF-α, IL-10, MIG, IL-6, IP-10, M-CSF, G-CSF, GM-CSF, and IFN-α2, that can be used as good predictors of ICU requirement at 4-6 DfSO.

## Introduction

Severe acute respiratory syndrome coronavirus 2 (SARS-CoV-2) is a novel betacoronavirus that emerged in December 2019 in Wuhan (China) and resulted in the current pandemic of coronavirus disease 2019 (COVID-19) (1). By September 2021, more than 218 million people have been diagnosed with COVID-19, and approximately 4,5 million people have died during the pandemic (2). In most cases, the disease course is mild (with or without pneumonia); however, dyspnea, hypoxia, and greater than 50% lung involvement can develop in severe cases, possibly leading to acute respiratory distress syndrome (ARDS), multiple organ failure and death (3). Mortality in COVID-19 patients admitted to the intensive care unit (ICU) has exceeded 35.5% (4).

The host immune response to SARS-CoV-2 appears to play a critical role in the pathogenesis and progression of COVID-19 (5); the response is initiated when SARS-CoV-2 enters alveolar epithelial cells through ACE2 (6) (80% of ACE2-expressing cells) or through AXL (7) or CD147 (8) receptors. After internalization, the virus triggers the canonical response of the innate immune system *via* interaction with pattern-recognition receptors (PRRs) expressed by epithelial cells, macrophages and dendritic cells, with subsequent massive proinflammatory cytokine release and an enhanced cellular response aimed at preventing viral replication (9). Serum concentrations of proinflammatory cytokines strongly correlate with disease outcome and were increased in patients with severe disease (10). In severe cases, induced expression of inflammatory cytokines (especially IL-6, TNF-α) can shift from local to systemic inflammation (5) through dysregulation of immune pathways (9) and immune cell distribution (lymphopenia, T-cell exhaustion, increasing counts of macrophages and neutrophils) (11–13).

It is supposed one of the main causes of such hyperinflammation and the development of serious complications during COVID-19 is the delayed or impaired type I IFN response as the first line of antiviral defense (14). Among possible explanations, genetic factors (15), autoantibodies against type I IFNs (16) and viral immunosuppressive mechanisms (5) have been discussed (17). Nevertheless, there are contradictory data (17) regarding the kinetics of early type I IFN responses.

In addition to IFNs, there has been extensive research on prospective inflammation markers in COVID-19 patients through measurement of increased serum levels of cytokines, chemokines and growth factors (18–21). Moreover, several immunological cytokine profiles based on disease severity (IL-6, TNF-α, IL-8, IL-10, G-CSF) (19, 21) have been defined, as have several patient demographic characteristics, including age (IL-6, IL-8, TNF-α) (19, 22), sex (IL-6, IL-18, IL-7) (23, 24) and the presence of noninfectious comorbidities (IL-6, IL-8, TNF-α) (19, 25). Some of these factors have been proposed for use as predictors of severity and pharmacologically relevant targets in anti-cytokine therapy (IL-6, IL-10, TNF-α, IFN-γ) (9, 20). Clinical trials are underway, but there are no satisfactory data on their effect thus far (14). To achieve appropriate implementation of new therapeutic agents for COVID-19 treatment, it is necessary to determine possible immunopathological mechanisms of action to predict complications and to determine the proper time frame in which interventions can be safely performed. Thus, longitudinal analysis performed within short time intervals can expand our understanding of the initial stages of disease and identify early markers to act as predictors of disease severity.

This study represents a comprehensive analysis of immune markers (46 cytokines and Ig A, M, G antibodies) in peripheral blood in a Moscow (Russia) cohort of 444 COVID-19 patients. The aim of our research was to investigate the dynamics of cytokines and antibodies in a general sample. We found early changes in cytokine levels (during the first three days from symptom onset) between patient groups with different disease severity. Moreover, we identified some immune signatures associated with sex, age and comorbidities in COVID-19 patients. All these findings will be useful for the prognosis of COVID-19 severity and the development of different therapeutic strategies.

## Materials and Methods

### Study Design and Participants

**Figure 1:**
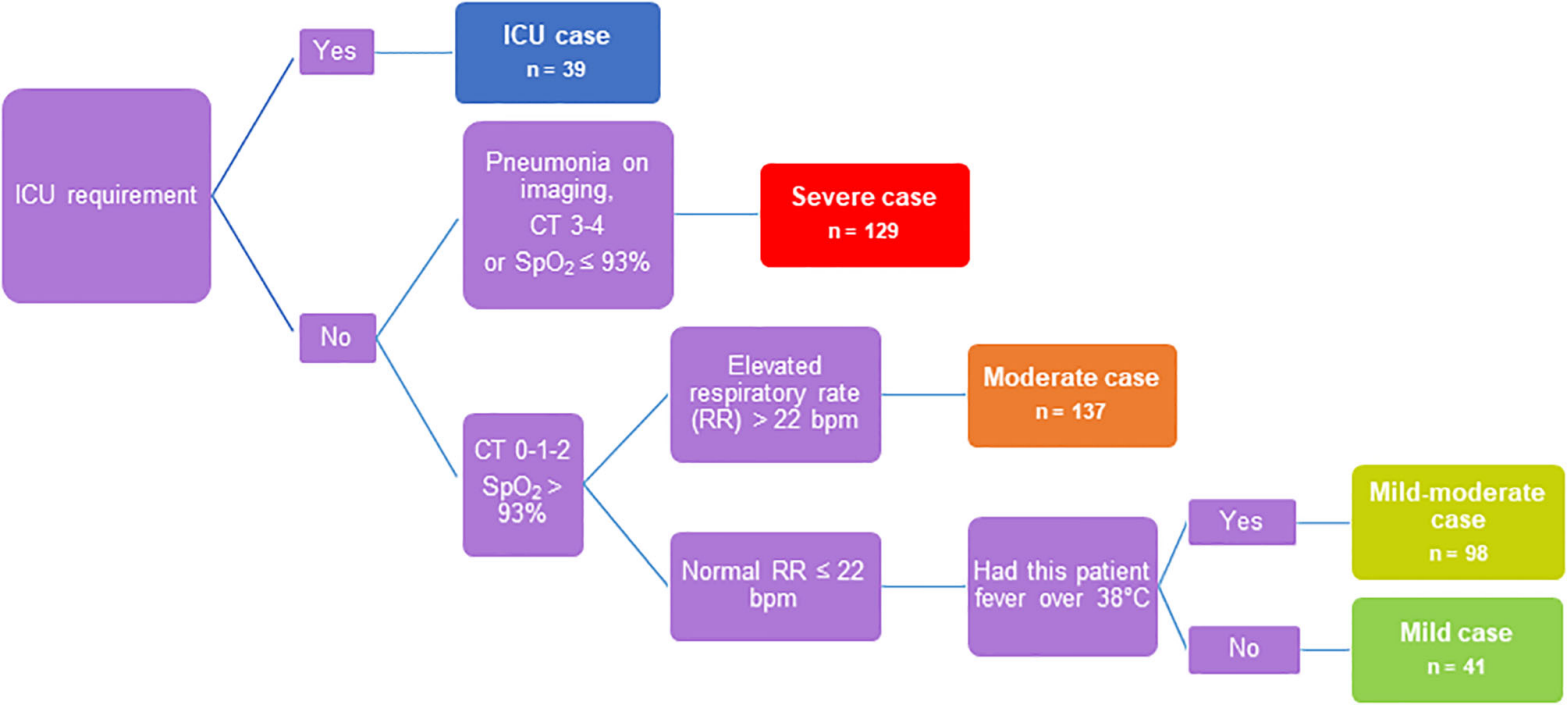
Flow chart to determine disease severity.

In this study, serum samples were obtained from adult COVID-19 patients seen at Clinic of Infectious Diseases №1 of Moscow Healthcare Department during the first wave of COVID-19 incidence from May to July 2020. A cohort of 444 COVID-19 patients was classified into 5 severity groups based on clinical characteristics and guidelines in the management of COVID-19 (**Figure 1** **)** (26). The main criteria were chest imaging (computed tomography (CT) score: degree of involvement ≤50% - score 1-2, >50% score 3-4), saturation of oxygen (SpO_2_), respiratory rate and fever. The group of ICU patients was separated due to the requirement of intensive care unit therapy (n=39): severe COVID-19 patients (n=129) - CT score 3-4, SpO_2_ ≤ 93%, respiratory rate ≥22 breaths/min; moderate COVID-19 patients (n=137) - CT score 0-1-2, SpO_2_>93%, respiratory rate ≥22 breaths/min; mild-moderate COVID-19 patients (n=98) - CT score 0-1-2, SpO_2_>93%, respiratory rate <22 breaths/min, body temperature (t) ≥38°C; and mild COVID-19 patients (n=41) - CT score 0-1-2, SpO_2_>93%, respiratory rate <22 breaths/min and t <38°C. Some patients required oxygen therapy, which included nonmechanical and mechanical ventilation with oxygen. The clinical characteristics of all patients are summarized in **Table S1**. Twenty-seven ICU patients developed critical illness during hospitalization and died (69%), and one patient with severe disease died without being in the ICU. Ethical approval for all patients was granted by the local Ethics Committee of Clinic of Infectious Diseases №1 of Moscow Healthcare Department, Moscow, Russia: Protocol No. 2/a from 11 May 2020. Informed consent was obtained, and a questionnaire (**Table S3**) was completed for all enrolled patients. Blood and nasopharyngeal swab samples from each COVID-19 patient were drawn three times during hospitalization: on the admission day, after 4 days (median with 95% CI 3-8) and on the discharge day (median - 12 days with 95% CI 7-23). Sera were collected and stored at -30°C until use. Serum samples of healthy donors (HD; n=66, **Table S1**) were obtained from N.F. Gamaleya National Research Center Biobank, which was collected in Russia during the first half of 2019 before the COVID-19 pandemic, frozen and stored at -80°C without other freeze-thaw cycles.

A cohort of 62 COVID-19 patients named “SCG” (seroconversion group) was selected from among all 444 patients according to antibody assay results. These patients were IgM+IgA positive and IgG negative at the first blood sampling point (at admission day) and became IgG positive at the second sampling point. SCG included mild (n=7 or 11%), mild-moderate (n=14 or 23%), moderate (n=22 or 35%), severe (n=15 or 24%) and ICU (n=4 or 6%) cases.

### SARS-CoV-2 RNA Detection

Nasopharyngeal swabs were tested using commercial reagent kits for determining the presence of SARS-CoV-2 RNA by real-time PCR: “SARS-CoV-2 FRT” manufactured by N.F. Gamaleya National Research Center, Russia and “SARS-CoV-2/SARS-CoV” manufactured by DNA Technology, Russia. Testing of samples was carried out in accordance with the manufacturer’s instructions.

### Antibody Detection

Levels of IgG antibodies against SARS-CoV-2 antigens (N-protein, RBD and S1) were estimated by xMAP SARS-CoV-2 Multi-Antigen IgG Assay and xMAP SARS-CoV-2 IgG Control Kit (Luminex Corp.) using the serum samples of 223 COVID-19 patients (mild (n=20), mild-moderate (n=41), moderate (n=71), severe (n=60), ICU (n=31) according to the manufacturer’s instructions. Acquisitions were performed using a MAGPIX instrument operated with xPONENT software version 4.2 (Luminex Corp.). Assay’s sensitivity and specificity characteristics: for ≤7; 8-14; >14 days from symptom onset positive percent agreement was 71.1% (55–83% 95% Cl); 80.0% (58–92% 95% Cl); 98.1% (90–100% 95% Cl) respectively, and negative percent agreement was 100% (99%-100% 95% Cl). Serum IgM and IgA in samples from all 444 COVID-19 patients were measured using a COVID-19 ELISA IgM+IgA kit (Vircell) following the manufacturer’s instructions. Optical density measurements were performed using a Multiscan FC microplate photometer operated with Skanit Software version 4.1 (Thermo Scientific). Assay’s sensitivity in patients 5 days after a positive PCR result was 88%, and specificity in samples from healthy pre-pandemic donors was 99%.

### Cytokine Analysis

Serum samples frozen and stored at -30°C without other thawing were tested for simultaneous quantification of sCD40L, EGF, eotaxin, FGF-2, FLT-3L, fractalkine, G-CSF, GM-CSF, GRO-α, IFN-α2, IFN-γ, IL-1α, IL-1β, IL-1RA, IL-2, IL-3, IL-4, IL-5, IL-6, IL-7, IL-8, IL-9, IL-10, IL-12 (p40), IL-12 (p70), IL-13, IL-15, IL-17A, IL-17E/IL-25, IL-17F, IL-18, IL-22, IL-27, IP-10, MCP-1, MCP-3, M-CSF, MDC, MIG, MIP-1α, MIP-1β, PDGF-AA, TGF-α, TNF-α, TNF-β and VEGF-A with MILLIPLEX MAP Human Cytokine/Chemokine/Growth Factor Panel (Merck-Millipore). All assays were performed according to the manufacturer’s protocol for serum samples, utilizing recommended sample dilutions and standard curve concentrations (Merck-Millipore). Acquisitions were performed using a MAGPIX instrument operated with xPONENT software version 4.2 (Luminex Corp.).

### Statistical Analysis

Data were analyzed using GraphPad Prism software version 8.0.2. All datasets were tested for a normal distribution using the Shapiro–Wilk normality test. As all normality tests were negative, all data sets were compared using either nonparametric two-tailed Mann–Whitney tests, Kruskal-Wallis test with Dunn’s multiple comparison test, or the Wilcoxon test, as appropriate. The prognostic validity of the cytokine model (value) was evaluated by analysis of the ROC curve and was measured using the AUC. Differences were considered significant at *p<*0.05 (*p<0.05; **p<0.01; ***p<0.001; ****p<0.0001). Spearman’s rank correlation tests were used to reveal the association between cytokine levels and were carried out with the Hmisc package (ver. 4.4.2) and visualized with the corrplot package (ver. R 0.84). Other graphs were generated using GraphPad Prism (ver. 8.0.2.).

## Results

### Patient Clinical and Immunological Characteristics

To determine patterns and predictors of COVID-19 severity during the immune response to SARS-CoV-2, we focused our research on the dynamics of serum biomarker levels (antibodies, cytokines, chemokines and growth factors) in COVID-19 patients. We established a cohort (characterized in **Table S1**) of 444 clinically diagnosed COVID-19 patients admitted to the Clinic of Infectious Diseases №1 in Moscow, Russia. The criteria for the inclusion of patients in this study were the presence of a positive PCR test and/or positive result of anti-SARS-CoV-2 antibodies (Ab) assay (IgM+IgA detection). Number of subjects who were both COVID-19 PCR**^+^** and SARS-CoV-2 Ab positive - 290 (65%), COVID-19 PCR**^+^** and SARS-CoV-2 Ab negative – 8 (2%), COVID-19 PCR**^-^** and SARS-CoV-2 Ab positive – 146 (33%). Disease severity (mild, mild-moderate, moderate, severe and ICU patients) was identified according to guidelines for clinical management of COVID-19 (26) (the flow chart of disease severity determination is depicted in **Figure 1**). The disease severity was determined as the most severe degree of disease during observation period in hospital. Briefly, our cohort was characterized by a median age of 60 years, with a slight quantitative preponderance of females compared with males (56% and 44%, respectively), a median hospitalization period of 12.5 days, an in-hospital mortality rate of 6% and a median disease course from symptom onset to discharge of 21 days.

By analyzing levels of antibodies against SARS-CoV-2, we found that the humoral immune response in our sample generally developed according to a fairly standard scenario for COVID-19 (27). Antibodies of all three classes, appearing in some patients already in the first week of the disease, were significantly increased in general by the end of the second to the beginning of the third week of infection, and total IgM+IgA appeared slightly earlier than IgG (**Figure S1**).

To assess COVID-19 severity risk factors (males, 60+ years, comorbidities – obesity, diabetes) (3), we performed cytokine profiling for our cohort, as distributed by sex, by age (<60 years, 60+) and the presence of noninfectious comorbidities. These results are described in the Supplementary materials (**Figures S2, S3** and **Table S2**).

### Cytokine Dynamics in COVID-19-Patients

**Figure 2:**
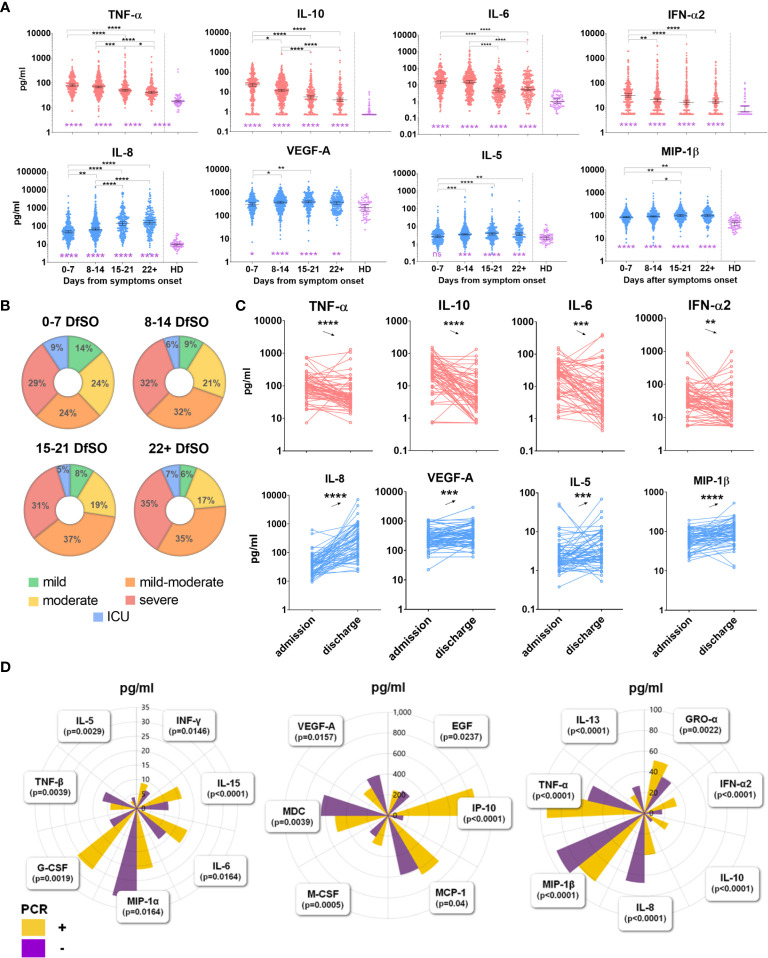
**(A)** Dynamics of serum cytokine levels during the disease course in the general COVID-19 patient cohort. Dynamics were measured in terms of days from symptom onset. All time points of sample collection from 444 patients were stratified into four intervals of 7 days starting from symptom onset. Healthy donors (HD) include 66 persons. Dots show individual measurements, and lines present medians with 95% CIs. Light red indicates “early” cytokines; light blue indicates “late” cytokines. Groups were compared by the Kruskal-Wallis test with Dunn’s *post hoc* test. *p<0.05, **p<0.01, ***p<0.001, ****p<0.0001. **(B)** Distribution of patients with different severity on each time interval of dynamics. **(C)** Comparison of serum cytokine levels at admission and discharge in “SCG” patients. For comparison analysis, a nonparametric Wilcoxon test was used, *p<0.05, **p<0.01, ***p<0.001, ****p<0.0001. **(D)** Comparison of cytokine levels between two cohorts of COVID-19 patients with different PCR test results on the admission day. In PCR “+” group n=298, in PCR “-” group n=146 (data for IL-27 not shown due to its serum level is out of range of plots). The groups were compared by a two-tailed Mann–Whitney U-test for nonparametric comparison.

Longitudinal cytokine analysis was performed for all patients to determine general kinetic patterns in the COVID-19 immune response. Time points of blood sample collection were stratified into four intervals of 7 days starting from symptom onset. Patients of all severity groups were included in each time interval of dynamics equally **(** **Figure 2B** **)**. Our results allowed us to identify statistically significant changes in 27 cytokines (**Figure 2A** and **Figure S4**). Concentrations of fifteen cytokines (including proinflammatory and anti-inflammatory cytokines, chemokines and growth factors) were the highest on 0-7 days from symptom onset (DfSO) interval, and then declined steadily after 7 DfSO (IFN-α2, IL-10, IL-27, GRO-α, MCP-1, G-CSF, M-CSF) or after 14 DfSO (IFN-γ, TNF-α, IL-6, IP-10, IL-15, IL-18, MIG, GM-CSF). These markers we considered “early” cytokines. The other group of cytokines included those that showed positive dynamics and increased from 0-7 DfSO to 15-21 or 22+ DfSO (IL-4, IL-5, IL-7, IL-8, MIP-1β, VEGF-A, sCD40L, FLT-3L, TNF-β, MDC, IL-13, PDGF-AA). This group we named as “late” cytokines.

Furthermore, we distinguished two phases of the disease depending on the result of the PCR test on the day of the patient’s admission to the hospital. As a result, all patients diagnosed with COVID-19 were divided into 2 cohorts: one in which it was still possible to detect virus from the nasopharynx by PCR (PCR “+”, n=298); the other included patients in whom the virus was no longer detected but who still exhibited symptoms of the disease (PCR “-”, n=146). We compared serum cytokine levels in these two cohorts. A total of 21 cytokines were revealed, which concentrations differed between the two cohorts (**Figure 2D**). The results for the majority of cytokines confirmed the findings for dynamics in the general cohort described above. For instance, serum levels of IFN-α2, IL-6, IL-10, IP-10, and M-CSF, which tended to decrease (**Figure 2A**), were also higher in the PCR “+” cohort than in the PCR “-” cohort (**Figure 2D** **)**. Conversely, serum levels of IL-8, MIP-1β, VEGF-A, which tended to increase (**Figure 2A**), were also higher in the PCR “-” cohort than in the PCR “+” cohort (**Figure 2D** **)**.

To determine which of the cytokines were elevated in the acute phase and which remained elevated on the discharge day (recovery phase), we selected a group of COVID-19 patients according to their seroconversion data, “SCG” patients. These patients were IgM+IgA positive and IgG negative at the first blood sampling point (at admission day), became IgG positive at the second sampling point and on the day of discharge.

Comparative analysis of cytokine levels in “SCG” patients revealed that twelve of them were elevated on the admission day compared with the discharge day (**Figure 2C** and **Figure S5**). All of them were confirmed by general cohort dynamics as “early” cytokines, which tended to decrease after 7 or 14 DfSO (**Figure 2A** and **Figure S4**). Conversely, serum levels of thirteen cytokines remained elevated on the discharge day compared to admission (**Figure 2C** and **Figure S5**); ten of them displayed the dynamics of “late” cytokines. A summary of cytokine level changes revealed by the three approaches described above (dynamics in the general cohort, based on PCR and IgG seroconversion) is shown in the **Table 1**.

**Table 1:** Summary of cytokine level changes revealed by the three approaches.

Cytokines	Dynamics in general cohort	PCR “+” *vs* PCR “-”	Dynamics in SCG patients	Cytokines	Dynamics in general cohort	PCR “+” *vs* PCR “-”	Dynamics in SCG patients
**G-CSF**	**“early” cytokines↘** ^**^ **to 15-21 DfSO**	**↘**	**↘**	**IL-8**	**“late” cytokines↗** ^*^ **to 15-21 DfSO**	**↗**	**↗**
**GRO-α**				**MDC**			
**IFN-α2**				**MIP-1β**			
**IL-10**				**VEGF-A**			
**IP-10**				**TNF-β**			
**M-CSF**				**IL-5**			
**IFN-γ**				**IL-13**			**ns**
**IL-6**				**IL-4**		**ns**	**↗**
**IL-15**				**FLT-3L**			
**TNF-α**				**PDGF-AA**			
**IL-27**				**sCD40L**			
**MCP-1**			**ns**	**IL-7**			**ns**
**IL-18**		**ns** ^***^	**↘**	**EGF**	**ns**	**↗**	**↗**
**GM-CSF**			**ns**	**MIP-1α**			**ns**
**MIG**				**Eotaxin**		**ns**	**↗**

* ↗, cytokine level significantly increased, ** **↘**, cytokine level significantly decreased, *** ns, not significant.

We performed correlation analysis for “SCG” patients to identify correlation relationships between cytokine levels at admission and discharge (in the acute and recovery phases). Multiple correlations were found among all cytokines (**Figure S6** **).** On the last day of hospitalization, we identified both repeats of the data of the first correlogram and completely new correlation pairs. IFN-α2, the main cytokine of innate immunity, showed a strong correlation with the primary acute-phase proinflammatory cytokines TNF-α (r=0.6, *p<*0.0001), IL-1β (r=0.6, *p<*0.0001) and IL-15 (r=0.7, *p<*0.0001). These connections may be illustrated by known data for the beginning of the antiviral response (21, 28). Another example is a “correlation triangle” between IL-6, IL-10 and TNF-α. IL-10, which is known as a suppressor in the initiation phase of inflammation during COVID-19 and correlated strongly with proinflammatory IL-6 (r=0.5, *p<*0.0001) and TNF-α (r=0.6, *p<*0.0001), probably performing an immune-inhibitory mechanism as a negative feedback inflammation loop (29).

### Differences in Cytokine Levels Depending on COVID-19 Severity

We examined cytokine levels in patients with different degrees of COVID-19 severity. For this, maximum cytokine levels during hospitalization for each patient were compared.

**Figure 3:**
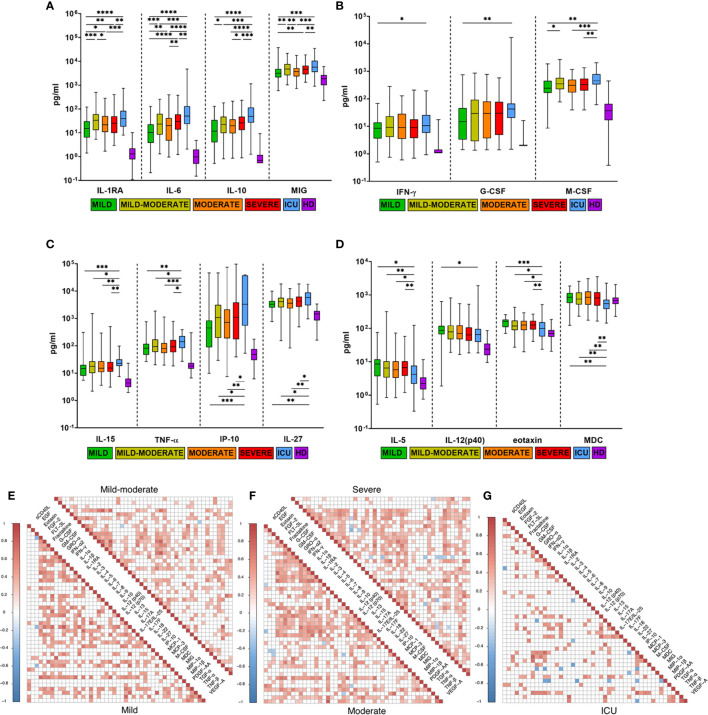
Comparison of cytokine levels in COVID-19 patients with different disease severities. **(A–D)** Comparison of maximum cytokine levels from each patient (n=444) divided into five severity groups: mild (n=41), mild-moderate (n=98), moderate (n=137), severe (n=129), ICU (n=39), HD (healthy donors, n=66). Boxes and whiskers represent medians with 95%CIs. Groups were compared by a two-tailed Mann–Whitney U-test for nonparametric comparison. *p<0.05, **p<0.01, ***p<0.001, ****p<0.0001. Compared with HD, all groups of COVID-19 patients had significantly higher levels of all examined cytokines (*p<*0.05), except MDC. **(E–G)** Cytokine correlation matrices for COVID-19 patients on the admission day (0-7 DfSO): mild (n=30), mild-moderate (n=54), moderate (n=59), severe (n=69) and ICU (n=22). Colors indicate Spearman correlation coefficients (*p<*0.05), and colorless squares indicate *ns* (not significant) values.

In addition to the cytokines already known to be associated with disease severity in COVID-19 (IL-6, IL-10, IL-27, IL-15, G-CSF, M-CSF, IP-10, MIG, TNF-α, IL-1RA) (19, 21), which were higher in the patients in our cohort with more severe COVID-19, cytokines with significantly lower concentrations in ICU patients than in others were detected: IL-5, MDC, eotaxin, and IL-12(p40). (**Figure 3** **)**. We divided all of these cytokines into three groups: the first group, IL-1RA, IL-6, IL-10 and MIG, showed increased expression together with disease severity (**Figure 3A**). The second groups included IL-15, IL-27, IP-10, TNF-α, M-CSF, G-CSF and IFN-γ, and levels were significantly higher in ICU patients than in other severity groups (**Figures 3B, C** **)**. The third group of cytokines included eotaxin, MDC, IL-5 and IL-12 (p40), and their serum concentrations were significantly lower in the ICU group than in the other groups (**Figure 3D**).

To evaluate the impact of disease severity on correlations between cytokine levels at the beginning of the disease (0-7 DfSO), we selected patients based on one parameter: the time from illness onset to hospitalization of no more than 7 days (n=234, selected from the general COVID-19 cohort). This new cohort included mild (n=30), mild-moderate (n=54), moderate (n=59), severe (n=69) and ICU (n=22) cases, and cytokine levels in blood at the first time point (admission day) were used for analysis in correlation matrices (**Figures 3E** **–G**). The mild group was characterized by the largest number of strong correlations (r>0.7) compared with the other groups. In general, the ICU group had a smaller number of high-positive (for most cytokines) but larger negative (for MIP-1β and MDC) correlations than the other groups, especially the mild group. IL-15 exhibited strong correlations in the mild group (0.67<r <0.8) with INF-α2, IP-10, G-CSF, M-CSF, and TNF-α (*p<*0.01); IL-10 also had a strong correlation with IL-6 (r=0.72, *p<*0.0001) and G-CSF (r=0.75, *p<*0.0001). For all severity groups, high correlations between TNF-α and IP-10 (0.7<r<0.8, *p<*0.01) were revealed. Some positive correlations between TNF-α and other cytokines (GM-CSF, GRO-α, IFN-α2, IFN-γ) were strengthened in the ICU group (**Figures 3E** **–G**).

**Figure 4:**
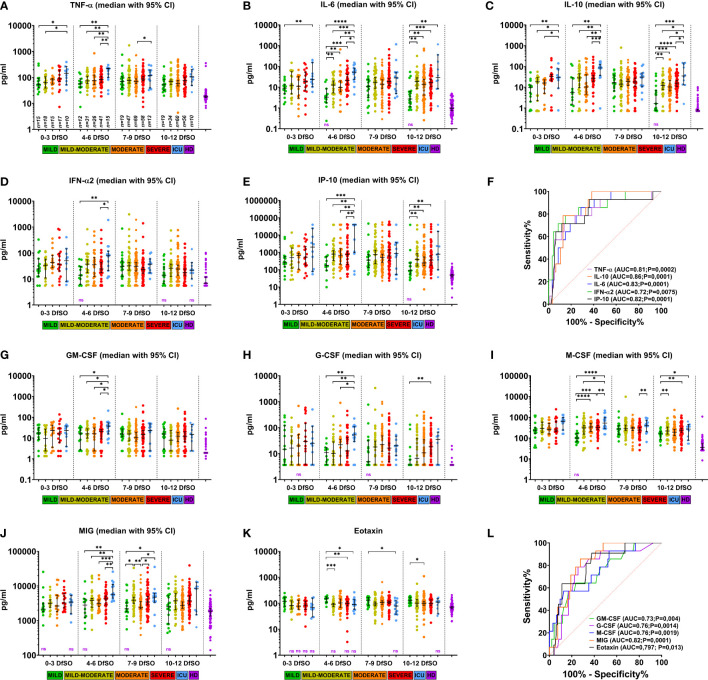
Dynamics of serum cytokine levels demonstrating differences between severity groups of COVID-19 patients in the first 12 days after illness onset. **(A–E)** and **(G–K)** show serum levels of TNF-α, IL-6, IL-10, IFN-α2, IP-10, GM-CSF, G-CSF, M-CSF, MIG, eotaxin in patients with different COVID-19 severities at days 0–3, 4–6, 7-9, and 10-12 DfSO. Dots show individual measurements, and lines represent medians with 95% CIs. Statistical analyses were performed with a two-tailed Mann–Whitney U-test for nonparametric comparison. All significant differences between severity groups are shown by asterisks: *p<0.05, **p<0.01, ***p<0.001, ****p<0.0001. For comparisons between any of severity groups and HD significant differences (p<0.05) are not shown and not significant differences provided as «ns» written in violet down of each plot. **(F, L)** demonstrate ROC curves of serum cytokine levels on 4-6 DfSO to predict ICU requirement during hospitalization. The ROC curve of eotaxin to predict mild forms.

Next, we determined the earliest time interval from the day of symptom onset on which differences in cytokine expression could be observed between patient severity groups. For this, dynamic analysis was performed for all cytokines depending on severity. Time points of sample collection were stratified into 4 periods with a short time interval of 3 days (the minimum time interval allowing us to populate all severity groups for comparison). As a result, we obtained a longitudinal period representing the first 12 DfSO. Overall, serum levels of 16 cytokines (TNF-α, IL-6, IL-10, IL-1RA, IFN-α2, IP-10, MIG, GRO-α, G-CSF, GM-CSF, M-CSF, IL-15, MCP-3, MCP-1, IFN-γ, eotaxin) differed significantly between severity groups within the first 12 DfSO (**Figure 4** and **Figure S7**).

All these cytokines showed differences in serum levels between severity groups on 4-6 DfSO time interval, with most differences being between the ICU group and all others. Some of them (TNF-α, IL-6, IL-10, IL-1RA, IL-15) **(** **Figures 4A–C** and **Figure S7A, E** **)** were elevated in the ICU group already within the first three DfSO.

Notably, there were 15 ICU patients with sample points in the 4-6 DfSO time interval. After 9 DfSO (median with 95% CI 8-13) these patients was transferred to the ICU. Among them 13 deaths (87%) occurred. Thus, the increasing in serum cytokine levels in these patients occurred before the time when they required ICU. Those patients were 70 years old (95% CI 63-82); 86% had cardiovascular diseases (CVDs).

Based on these results, we further analyzed whether these cytokines can be used as biomarkers to predict disease severity and mortality in COVID-19 and to determine patients for whom ICU therapy may be required further. Patients with blood sample time points of 4-6 DfSO were divided into ICU and non-ICU groups (all other severity groups), and ROC curves of each single cytokine were calculated using cytokine levels within 4-6 DfSO. The best result was an AUC of 0.86 (*p=*0.0001) for IL-10, followed by 0.83 (*p=*0.0001) for IL-6, 0.82 (*p=*0.0001) for MIG and IP-10, 0.81 (*p=*0.0002) for TNF-α, 0.76 (*p=*0.0014 and *p=*0.0019) for G-CSF and M-CSF, 0.73 (*p=*0.004) for GM-CSF, and 0.72 (*p=*0.0075) for IFN-α2 (**Figure 4F, L** **)**. Serum levels of eotaxin were also used in ROC analysis to distinguish the mild severity group from all others, and the AUC for eotaxin was 0.797 (*p=*0.013) (**Figure 4L**), suggesting that eotaxin is a potential biomarker of mild COVID-19.

## Discussion

In our study, we performed a comprehensive analysis of immune marker levels in the sera of 444 COVID-19 patients. The results revealed common patterns in disease course as well as differences according to sex, age, comorbidities and severity, which are previously unreported.

In most cases, the course of COVID-19 was characterized as mild (40%) and moderate (40%), without critical complications (3). In our cohort, only 9% of patients required ICU therapy. To identify immunological traits that may be common in the COVID-19 course, we performed longitudinal analysis of 46 cytokines for all patients. All cytokines with significant dynamic changes were conditionally divided into two groups: some tended to decrease (“early” cytokines), whereas others tended to increase (“late” cytokines) to 15-21 DfSO (**Figure 2A**, **Figure S4** and **Table 1**). Among the “early” cytokines, we found signatures indicating activation of the innate immune response (TNF-α, IL-6, IL-18, IL-27, IL-15, IFN-α2) and type 1 immunity (IFN-γ). These reactions characterize phases when virus recognition by epithelial or dendritic cells leads to the induction of innate host defenses and inflammatory responses, which in turn induce adaptive type 1 immunity (28). In addition, colony-stimulating factors (GM-CSF, G-CSF and M-CSF) and chemokines (GRO-α, IP-10, MIG, MCP-1) were detected as “early” cytokines. These factors are synthesized under the influence of proinflammatory cytokines responsible for the growth and activation of monocytes, macrophages, neutrophils and their attraction to damaged tissue, resulting in further inflammation (30). In contrast, the “late” cytokines characteristic of type 2 immunity (IL-4, IL-5, IL-13) (28) were increased after 7 DfSO, which may indicate enhancement of the humoral response, differentiation and proliferation of B-cells. Type 1 outcomes generate both cell-mediated and humoral responses that act synergistically, whereas type 2 outcomes generate humoral responses but actively suppress cell-mediated responses (31). Our results showed switching responses from type 1 to type 2 immunity after 7 DfSO in COVID-19 development; that is, similar to most infections, type 1 immunity is protective, whereas type 2 responses assist with the resolution of cell-mediated inflammation (31). VEGF-A, IL-8, PDGF-AA, and EGF were identified as “late” cytokines and may act as mediators of wound healing and tissue repair (32). IL-8 is a proinflammatory chemokine associated with the promotion of neutrophil chemotaxis and degranulation, and given the frequent neutrophilia observed in patients infected with SARS-CoV-2, it is possible that IL-8 contributes to COVID-19 pathogenesis (33). Regardless, as our results revealed a strong increase in IL-8 up to the discharge day, it may be possible that it plays a role in angiogenesis (34).

We identified the same cytokines associated with COVID-19 severity (IL-6, IL-1RA, IL-10, MIG, IL-15, G-CSF, M-CSF, IP-10, TNF-α, IL-27) (**Figures 3A** **–C**) as found in recent studies (18, 19, 22, 24, 35). In addition to these cytokines, we identified previously unreported cytokines with serum levels that were significantly lower in the ICU group than in the other groups, including MDC, eotaxin, IL-5 and IL-12(p40) (**Figure 3D**). It is unclear which role the decreased serum levels of these cytokines play in the ICU group. Correlation analysis (**Figures 3E** **–G**) showed that IL-5 and MDC had negative correlations (0.5<r <-0.4, *p*<0.05) with IL-8, IL-18, M-CSF and GRO-α, IFN-γ, IL-2, IP-10, respectively, only in the ICU group. IL-12 (p40) in the mild group had strong correlations with other factors of innate response, such as IL-1RA, IL-15, M-CSF, IFN-α2, GRO-α (0.4<r<0.65, *p*<0.05), whereas IL-12 (p40) maintained a high correlation only with IFN-α2 (r=0.6, *p*<0.01) in the ICU group, which may indicate dysregulation of innate immunity.

According to prevalent trends in COVID-19 research, several major demographic (age and sex) and clinical (noninfectious comorbidities) characteristics are associated with an increased risk of disease severity and mortality. There is increased risk of death for both sexes with advancing age, but at all ages above 30 years males have a significantly higher risk of death than females (23). In addition to the previously reported sex and age associated cytokines in COVID-19 (22–24) we identified some new ones. Among COVID-19 patients levels of IL-7, IP-10 and G-CSF were higher in male and MDC level was increased in female (**Figures S2A** **)**. Levels of eotaxin, sCD40L, IL-12 (p70), MDC, and PDGF-AA were higher in the younger group less (60 y.o.) (**Figure S2C, D** **).** As for comorbidities EGF and IL-13 were the most frequently occurring cytokines, which serum levels were lower in patients with any comorbidity compared to without it **(** **Table S2** **)**.

All cytokines described above, which were identified according to disease severity, can be used as severity biomarkers or as therapeutic targets for the prevention of poor outcomes of COVID-19 (9, 20). However, to achieve this goal, it is necessary to determine the time frames in which critical changes occur. In our study, we observed changes in cytokine levels between severity groups in the early stage of the disease (within the first 12 DfSO, **Figure 4** and **Figure S7**) and demonstrated differences in cytokine expression between severity groups within the first days of symptom onset. The factors identified are both proinflammatory and anti-inflammatory cytokines (TNF-α, IL-6, IL-15, IFN-γ, IL-10, IL-1RA), type I IFN (IFN-α), chemokines (GRO-α, IP-10, MIG, MCP-3, MCP-1) and growth factors (G-CSF, GM-CSF, M-CSF). All of them are involved in the innate immune response. Interaction of SARS-CoV-2 with the host immune system can cause hyperinflammation in critical cases at the very beginning of COVID-19 that was demonstrated in the 4-6 DfSO time interval, with the highest cytokine serum levels in the ICU group for all listed cytokines. Furthermore, the median of days of transferring to the ICU was 9 DfSO, which indicates that found cytokines can be used as predictors of COVID-19 severity. Based on ROC analysis, nine biomarkers (TNF-α, IL-10, MIG, IL-6, IP-10, M-CSF, G-CSF, GM-CSF, IFN-α2) were established as good early predictors for COVID-19 patients who may require ICU admission (**Figures 4F, L** **)**.

Our results show significantly higher levels of proinflammatory cytokines (TNF-α and IL-6) and anti-inflammatory cytokines IL-10 and IL-1RA in the severe and ICU groups within the first three days after illness onset (**Figures 4A–C** and **Figure S7A**). These cytokines have been suggested as biomarkers to predict the severity and mortality of COVID-19 patients (20, 36). On the one hand, it is thought that elevated serum levels of IL-10 and IL-1RA in COVID-19 patients act as anti-inflammatory or immunosuppressive cytokines to prevent hyperinflammation and are induced by the rapid accumulation of proinflammatory cytokines. On the other hand, high levels of IL-10 and IL-1RA in severe COVID-19 patients can be a signal of an overactive immune response, which may play a detrimental pathological role in COVID-19 severity. Indeed, a dramatically elevated serum level of IL-10 is a unique feature of the cytokine profiling of COVID-19 (29), and its expression by regulatory T cells (T_regs_) in severe COVID-19 has been demonstrated (37). IL-10, an inhibitory cytokine, not only prevents T cell proliferation but also induces T cell exhaustion (38). Previous studies of T cells in COVID-19 patients have reported signs of exhaustion (11, 39, 40) and lymphopenia (11, 40, 41). As blocking IL-10 function has been shown to prevent T cell exhaustion in animal models of chronic viral infection (42), thus anti-IL-10 therapy may be useful at the early stage of COVID-19. In addition, TNF-α can promote aged T cell apoptosis (43), which may contribute to lymphopenia in the severe course of COVID-19 in older patients (40).

There are conflicting data regarding the production of type I IFNs, which are important for antiviral innate immunity (21, 30, 44, 45). Some data show that IFN-α2 is produced in severe and critically ill patients (21, 30, 44) but that it is diminished in mild cases (30) or declines in patients with moderate disease (21). Another study found the type I IFN response to be high (between days 8-12) in mild-to-moderate cases but significantly reduced in more severe cases with a striking downregulation of IFN-stimulated genes (ISGs). It remains unclear whether reduced type I INF levels are present from the onset of disease (45). Such contradictory data can be explained by the absence or insufficient number of time points in longitudinal analysis, especially in the first days after illness onset.

In this study, we observed early expression (0-3 DfSO) of IFN-α2 regardless of severity and found its maximum value in the ICU group on 4-6 DfSO (**Figure 4D** **)**. In contrast, IFN-α2 was poorly expressed in the mild group, though its level remained significantly elevated in the other severity groups compared with the control for all 12-day periods. Thus, we did not find a delayed type I IFN response in critically ill patients, but the ICU group was characterized by rather strong expression of IFN-α2.

Overall, there were excessive levels of chemokines (GRO-α, IP-10, MIG, MCP-3, MCP-1) and growth factors (GM-CSF, G-CSF, and M-CSF) in the ICU group relative to the other severity groups on 4-6 DfSO (**Figure 4** **)**. High levels of chemokines and their receptors have been reported in COVID-19 patients (35, 46, 47). Chemokines attract neutrophils and macrophages (the main sources of proinflammatory cytokines) to the lungs and trigger further apoptosis of infected epithelial and endothelial cells (12, 13), and neutrophilia frequently develops in COVID-19 patients in the ICU (12, 35, 46, 48, 49). Activated neutrophils release a variety of injurious molecules, including neutrophil elastase and metalloproteases as well as other proteolytic enzymes, oxidants, and reactive nitrogen species (12, 50). Enhanced infiltration of the infection site by neutrophils and macrophages and its effects can result in damage to the pulmonary microvascular and alveolar barrier and cause vascular leakage and alveolar edema, which can lead to ARDS and other complications (12, 50).

The cytokine dynamics results presented in this study have some limitations. First, this study only characterized cytokine patterns in the peripheral blood but did not directly examine the respiratory tract or other possible sites of infection. Second, analysis of blood immune cells was not performed, and we did not explore which cells are the source of the cytokines detected. All assumptions regarding the relationship between the identified cytokines, chemokines, growth factors and blood cells are speculative and based on previous findings of immune patterns characterizing the course of COVID-19. Additionally, it is necessary to adjust for some limitations – both for the general cohort (the only medical clinic, genetic factors, pandemic period, etc.) and for the healthy donors cohort (in the HD cohort number of women is significantly higher than men, which differs from COVID-19 patients cohort). Nevertheless, the time frames and corresponding changes in cytokine responses found in our study can help in further longitudinal studies aimed at describing the dynamics of immune cells and their activation, differentiation, and possible dysregulation, which will deepen our understanding of COVID-19 pathology.

## Data Availability Statement

The raw data supporting the conclusions of this article will be made available by the authors, without undue reservation.

## Ethics Statement

The studies involving human participants were reviewed and approved by Ethics Committee of Clinic of Infectious Diseases №1 of Moscow Healthcare Department, Moscow, Russia: Protocol No. 2/a from 11 May 2020. Informed consent was obtained, and a questionnaire was completed for all enrolled patients.

## Author Contributions

VG and DK conceived of the study. LK, OB, IK, SS, and VB was responsible for the patient recruitment, collected the clinical data, and organized the patient specimen collection. DK, LP, EB, and EM performed the multiplex immunoassays, carried out the data analysis and drafted the manuscript. NK, ES, and ED performed the PCR verification analysis and specimen logistics, and completed and verified the database. VB and AP assisted in the specimen collection, data management, and statistical analysis. VG, AT, DL, and AG contributed to the study design and funding acquisition and provided valuable comments and suggestions. All authors critically revised the manuscript, contributed to the article and approved the submitted version.

## Funding

Study supported by the Ministry of Health of the Russian Federation, Government assignment number AAAA-A20-120113090054-6.

## Conflict of Interest

The authors declare that the research was conducted in the absence of any commercial or financial relationships that could be construed as a potential conflict of interest.

## Publisher’s Note

All claims expressed in this article are solely those of the authors and do not necessarily represent those of their affiliated organizations, or those of the publisher, the editors and the reviewers. Any product that may be evaluated in this article, or claim that may be made by its manufacturer, is not guaranteed or endorsed by the publisher.
