# Supplementary material for: A Deep Look Into COVID-19 Severity Through Dynamic Changes in Blood Cytokine Levels
Source: Front Immunol. 2021 Nov 9;12:771609. doi: 10.3389/fimmu.2021.771609 (PMC8630739; doi:10.3389/fimmu.2021.771609)
Supplement: Supplementary file 1 [file DataSheet_1.docx]

**Supplementary text**

**Humoral immune response in COVID-19 patients**

The development of the humoral immune response to SARS-CoV-2 is a highly dynamic process; thus, antibody kinetics are well described(1–3). In 52% of the patients in our cohort, total IgM+IgA against the SARS-CoV-2 nucleocapsid (N) and S1 were detected at 0-5 days from symptom onset (DfSO), and levels were significantly increased by 11-15 DfSO (**Fig. S1 А-Е**). In severe COVID-19 patients, the IgM+IgA response (comparing the maximum values from all sample points) was more intense than in the mild group (**Fig. S1 F**). IgG against the N-protein and RBD domain was detected earlier (in the second week from the onset of symptoms) than IgG against the S1 (on days 16-20) in all severity groups (**Fig. S1 A-E)**, and median values of the maximum levels of IgG antibodies to all three proteins in the ICU group were lower than those in the other groups (**Fig. S1 F**). Apparently, this is because more than half of the patients in the ICU group (69%) died before they had reached the IgG maximum production level.

The 100% cumulative seropositive rate for IgG, both to N-protein and to RBD (but not to S1), was observed for the mild patient group by days 16-20 and for the mild-moderate patient group by days 26-30. For more severe COVID-19 patients, it was not achieved during the observation period. The gradient of the 100% cumulative IgM+IgA seropositive rate for critical and severe cases was observed at 11-15 and 16-20 DfSO, respectively; for less severe forms, this phenomenon was observed only from the beginning of 4 weeks after symptom onset. The results of analysis of antibody (total anti-SARS-CoV-2 IgM+IgA; IgG to N-protein, S1 and RBD-domain) dynamics in the studied sample were generally consistent with the previously described trends of the humoral immune response in COVID-19(1–3). This fact gives confidence for the correct distribution of patients by severity and representativeness in our sample.

**Cytokine signatures in COVID-19 patients based on demographic signs and** **noninfectious comorbidity in medical history**

According to prevalent trends in COVID-19 research, several major demographic (age and sex) and clinical (noninfectious comorbidities) characteristics are associated with an increased risk of disease severity and mortality (4,5). Based on these data, comparative analyses of cytokine levels were performed between COVID-19 patients with and without any chronic disease, as well as for sex and age (<60 and 60+). For this, the maximum cytokine concentration values from three available sample points from patient were used for comparisons.

Relative to women, men with COVID-19 had their own pattern of "increased" cytokines: IL-6, IL-7 (*p*<0.01) and sCD40L, eotaxin, G-CSF, PDGF-AA, IL-18, IP-10 (*p*<0.05). At the same time, only the MDC level was higher in women (*p*<0.01) than in men (**Fig. S2 A**). Cytokine concentrations in both sex groups were significantly elevated (*p*<0.05) compared with those in healthy donors (HD).

In addition to the “classic” sex-associated COVID-19 markers proinflammatory cytokines IL-6(5), IL-18(6), ligand sCD40L(7) and PDGF-AA(7), which are higher in men, we found distinctively increased levels of the chemokine MDC in women, which might be due to its high expression by more active dendritic cells in women than in men(8). Additionally, levels of IL-7, IP-10 and G-CSF were higher in men, reflecting inflammatory processes that may develop during the COVID-19 acute phase(9).

Among well-known cytokine signatures associated with patient age(10), we found increased levels of M-CSF (*p*<0.05), IL-6, IL-27 (*p*<0.01) and MIG (*p*<0.0001) in the 60+ group; increased levels of EGF, eotaxin (*p*<0.01) and sCD40L, IL-12 (p70), MDC, and PDGF-AA (*p*<0.05) were detected in the younger group (**Fig. S2 C**). However, the same differences in the levels of five of these identified cytokines (IL-6, M-CSF, sCD40L, EGF, MIG) were found in HD group when comparative analysis was performed regarding age distribution (**Fig S2 D)**. Only the MDC level in COVID-19 patients <60 was not significantly different from that of the HD <60 group' other cytokine levels in the group of COVID-19 patients (<60 and 60+) were significantly elevated compared to those in the HD (<60 and 60+) group (*p*<0.05) respectively (data not shown).

Among demographic parameters in COVID-19 patient cohort studies, comorbidities are one of the main risk factors impacting the disease course(5). The proportion of patients with any comorbidities in the studied general sample was 73%. Following chronic disease were recorded: cardiovascular disease (CVD), including hypertension - 57%; chronic obstructive pulmonary disease (COPD) - 6%; bronchial asthma (BA) - 4%; diabetes mellitus (DM) types I and II - 17%, rheumatic disease (RD) - 5%; and obesity - 6% (**Table S2**). Our cohort also included patients with cancer (n=26), liver diseases (n=12) or neurological diseases (n=12). For the first group of patients, there were no statistically significant differences in levels of cytokines compared with the CM- cohort. For liver diseases and neurological diseases, the number of patients was insufficient for statistical analysis. It was noticed that with the development of severity, the proportion of patients with comorbidities increased: mild – 54%, mild-moderate – 74%, moderate – 69%, severe – 78% and ICU – 90%.

In our cohort, COVID-19 patients with any comorbidities (CM+) were found to have elevated serum levels of IL-1RA, IL-6, IL-27, MCP-1 and MIG compared to the cohort without comorbidities (CM-). In contrast, EGF, IL-13, IL-17A, TNF-β, and VEGF-A were lower than in CM- (**Fig. S2 B**). The cytokine concentrations of both groups were significantly elevated (*p*<0.05) compared to the healthy donor group (data not shown).

**Table S2** shows the results of comparing cytokine levels for each comorbidity separately. In addition, representative literary sources confirmed the relationship of the identified cytokines with the corresponding comorbidity and their possible function. In general, the most frequently occurring cytokines, with elevated serum levels in the CM- cohort, were EGF in CVD, BA+COPD, DM, RA, and obesity cohort and IL-13 in CVD, COPD, BA+COPD, and DM cohort comparison (**Fig. S3).**

**Supplementary Figures**

**
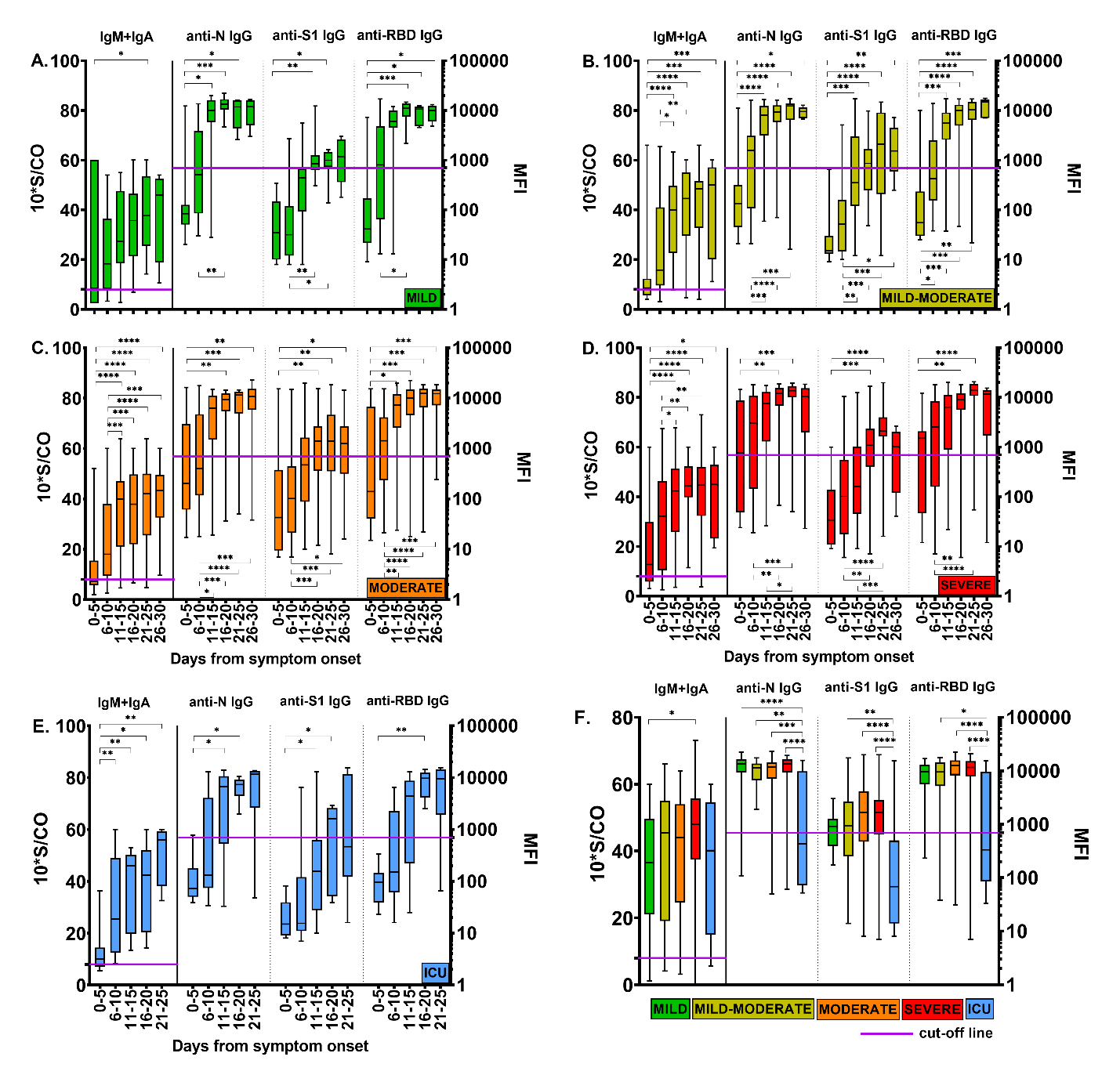
**

**Fig. S1. Dynamics of anti-(N+S1) total IgM+IgA and anti-N, anti-S1, and anti-RBD IgG in COVID-19 patient sera during the disease course.** The dynamics in different severity groups of patients. All time points of sample collection were stratified into 6 (5 for ICU) periods with an interval of 5 days **(A-E).** Comparison of maximum values of antibody measurements in each severity group. Box and whiskers represent medians and min/max values **(F).** IgM+IgA antibody levels are plotted along the left ordinate axis (10*S/CO) and IgG along the right (MFI). Statistical analyses were performed using the Kruskal-Wallis test with Dunn’s multiple comparison test (** p*<0.05, *** p*<0.01, **** p*<0.001, ***** p*<0.0001).

**
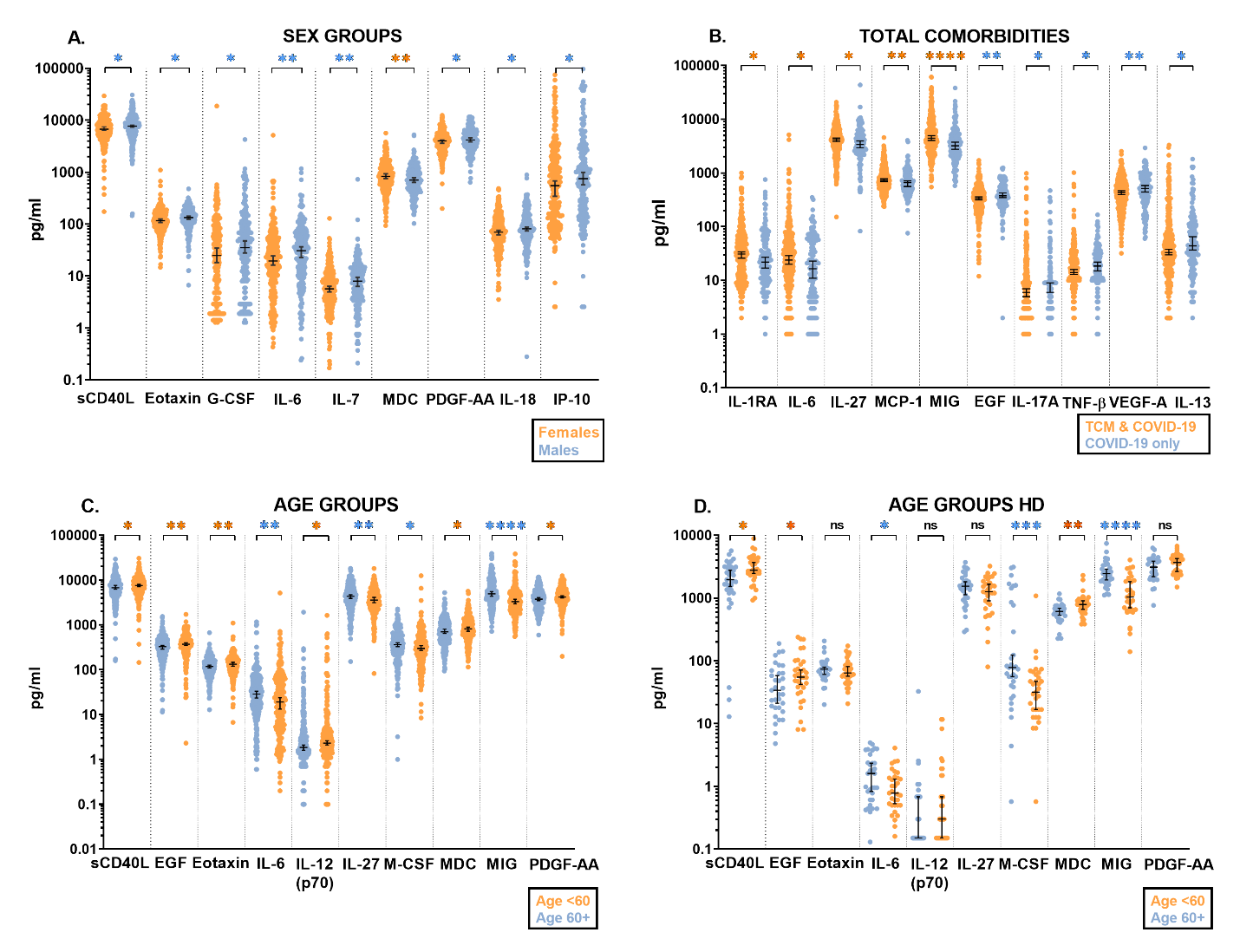
**

**Fig. S2. Comparative analysis of cytokine levels between COVID-19 patients regarding sex, age and chronic diseases.** **A.** Sex (orange – women, blue – men); **B.** comorbidities (orange – COVID-19 patients with comorbidities (CM+), blue - COVID-19 patients without comorbidities (CM-)). **C.** Age (orange – group less 60 y.o., blue – 60+ y.o. group); **D.** comparative analysis of HD regarding age (<60 and 60+). The maximum cytokine concentrations during the course of the disease were analyzed. Scatter plots indicating individual measurements (dots); lines represent medians with 95% CIs. Statistical analyses were performed using the Mann-Whitney univariate t-test *(* p*<0.05, *** p*<0.01, **** p*<0.001*, **** p*<0.0001, *ns-*not significant).


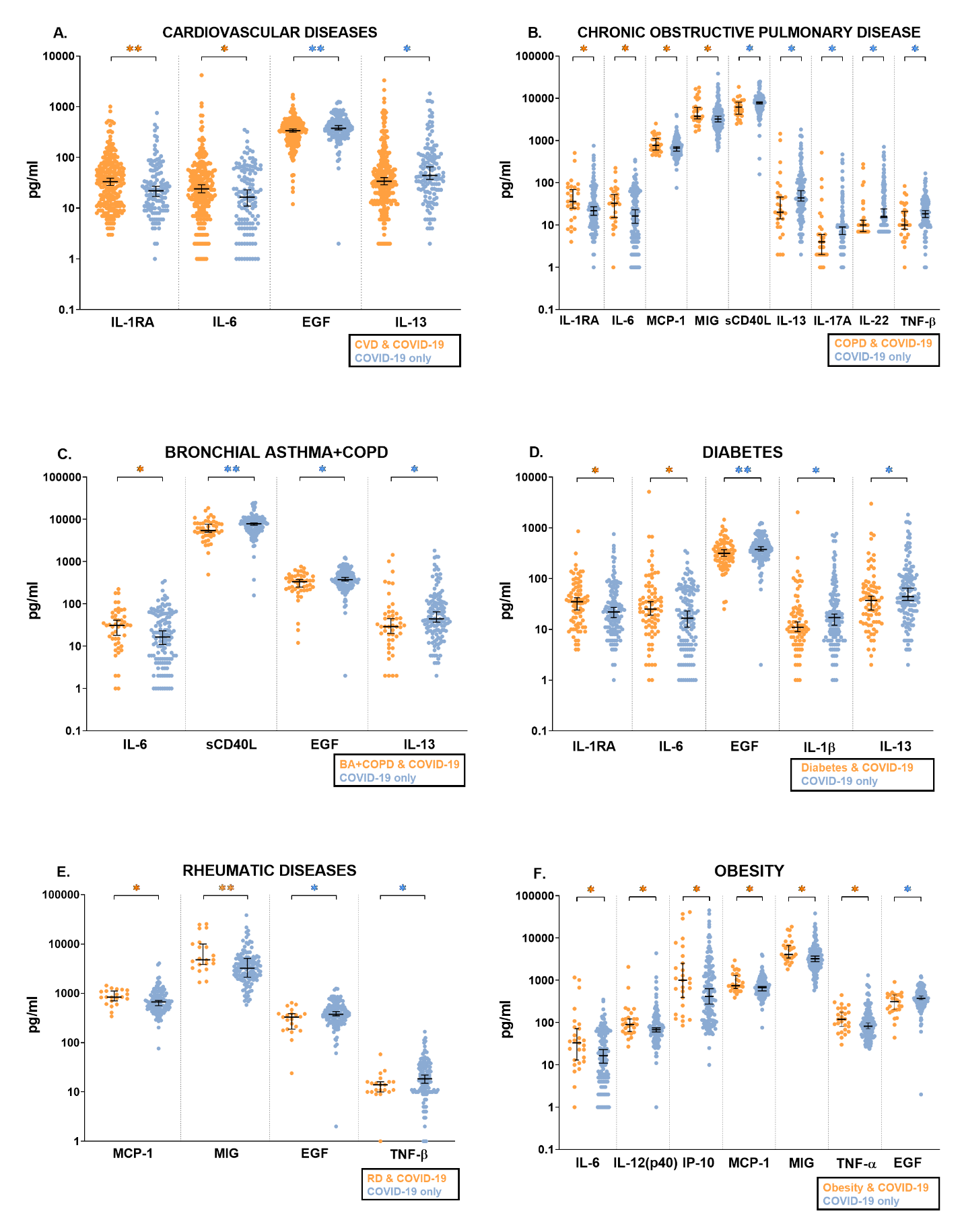


**Fig. S3. Comparative analyses of cytokine levels between COVID-19 patients regarding chronic diseases.** **A.** With or without cardiovascular diseases (CVDs); **B**. with or without chronic obstructive pulmonary diseases (COPD); **C.** with or without bronchial asthma+COPD; **D.** with or without diabetes; **E.** with or without rheumatic disease; **F.** with or without obesity. Lines represent medians with 95%CIs. Statistical analyses were performed using the Mann-Whitney univariate t-test *(* p*<0.05, *** p*<0.01, ***** p<0.001, ***** p*<0.0001, *ns-*not significant).

**
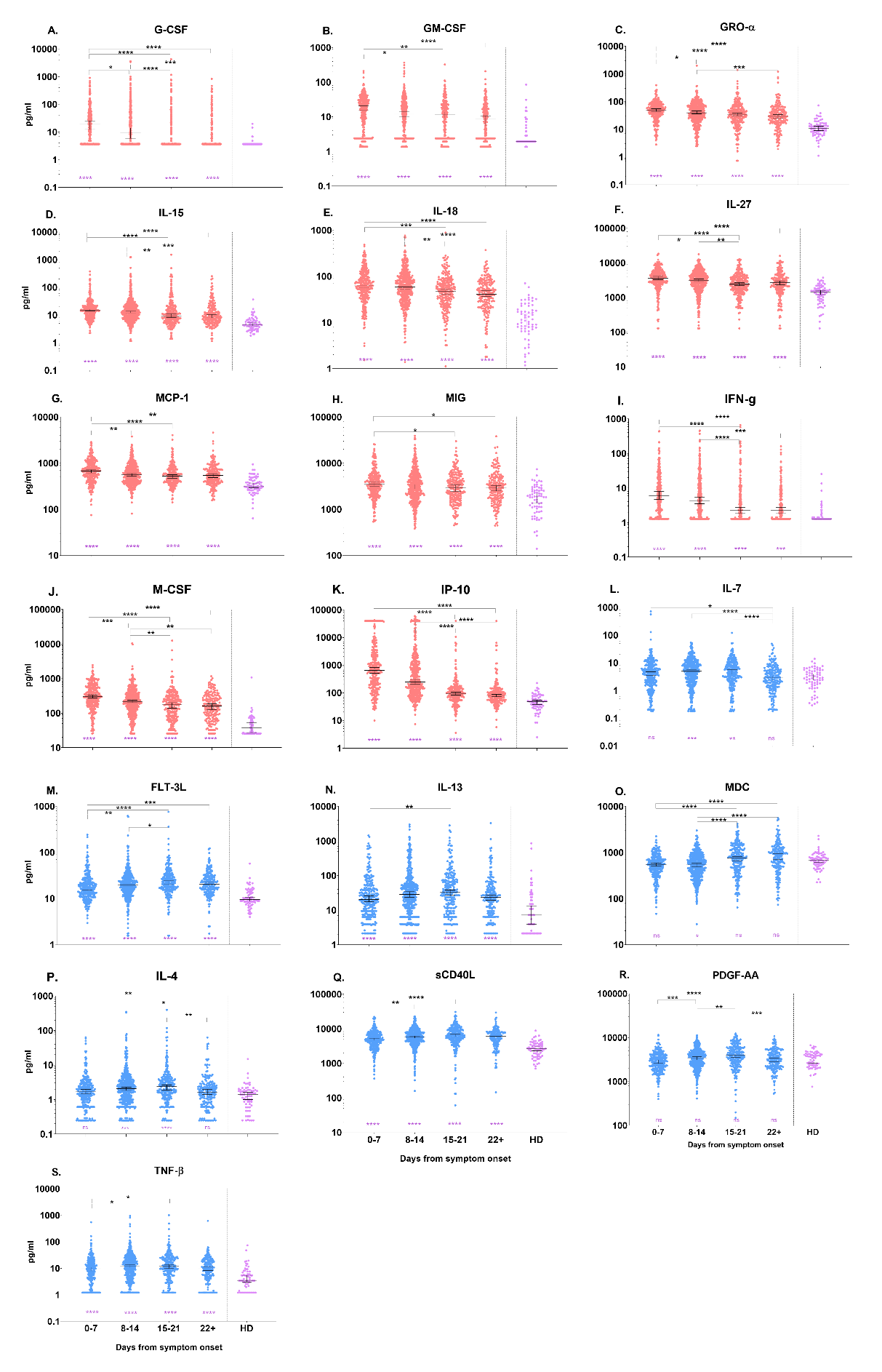
**

**Fig. S4. Dynamics of serum cytokine levels during the disease course in the general COVID-19 patient cohort.** Dynamics were measured in terms of days from symptom onset. All time points of sample collection from 444 patients were stratified into four intervals of 7 days starting from symptom onset. Healthy donors (HD) include 66 individuals. Dots show individual measurements; lines present medians with 95% CIs. Light red indicates “early” cytokines; light blue indicates “late” cytokines. Groups were compared using the Kruskal-Wallis test with Dunn’s post hoc test*. *p<*0.05*, **p<*0.01*, ***p<*0.001*, ****p<*0.0001*.*

**
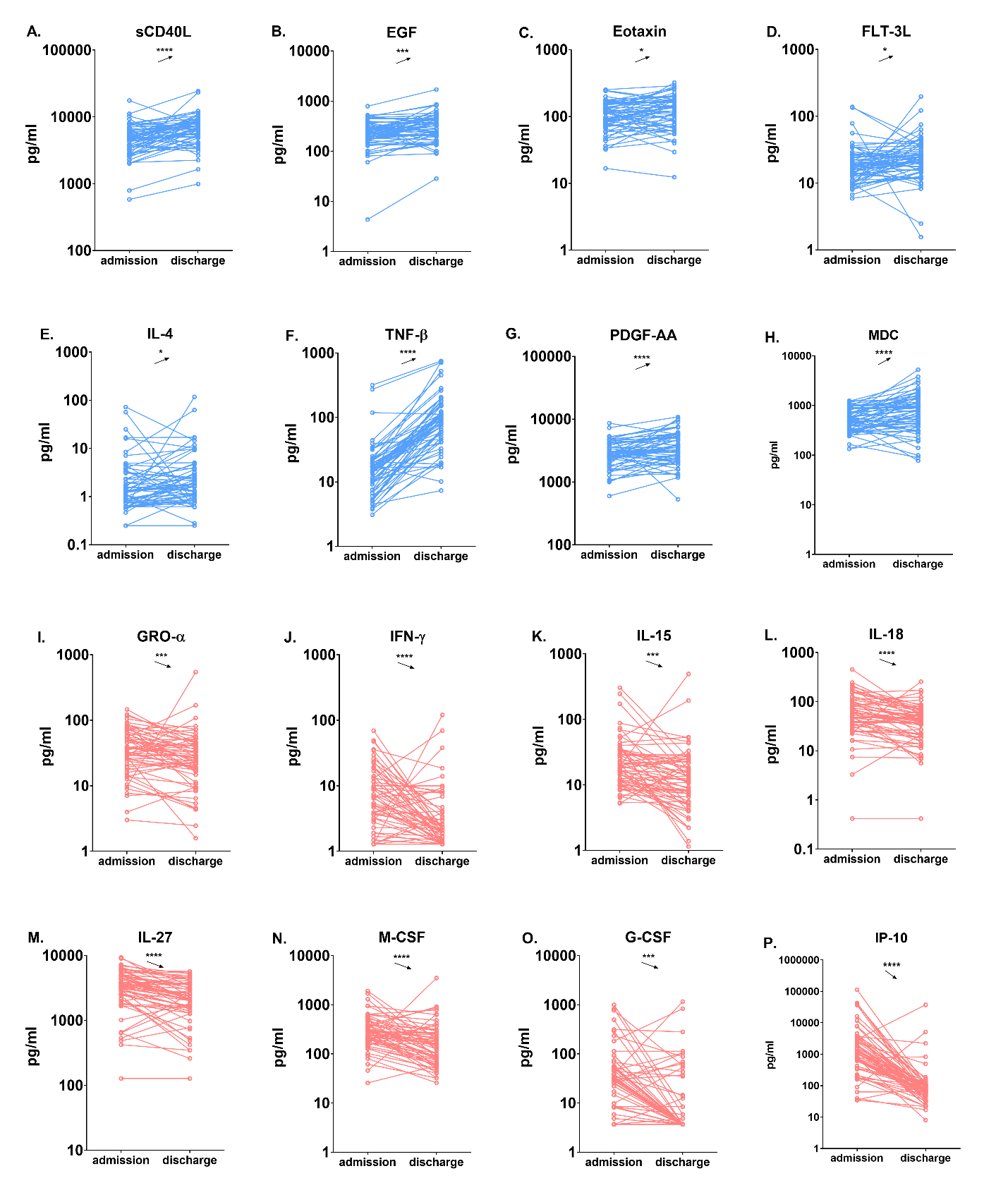
**

**Fig. S5. Comparison of serum cytokine levels at admission and discharge in “SCG” patients.** For comparison analysis, a nonparametric Wilcoxon test was used, **p<*0.05*, **p<*0.01*, ***p<*0.001*, ****p<*0.0001*.*

**
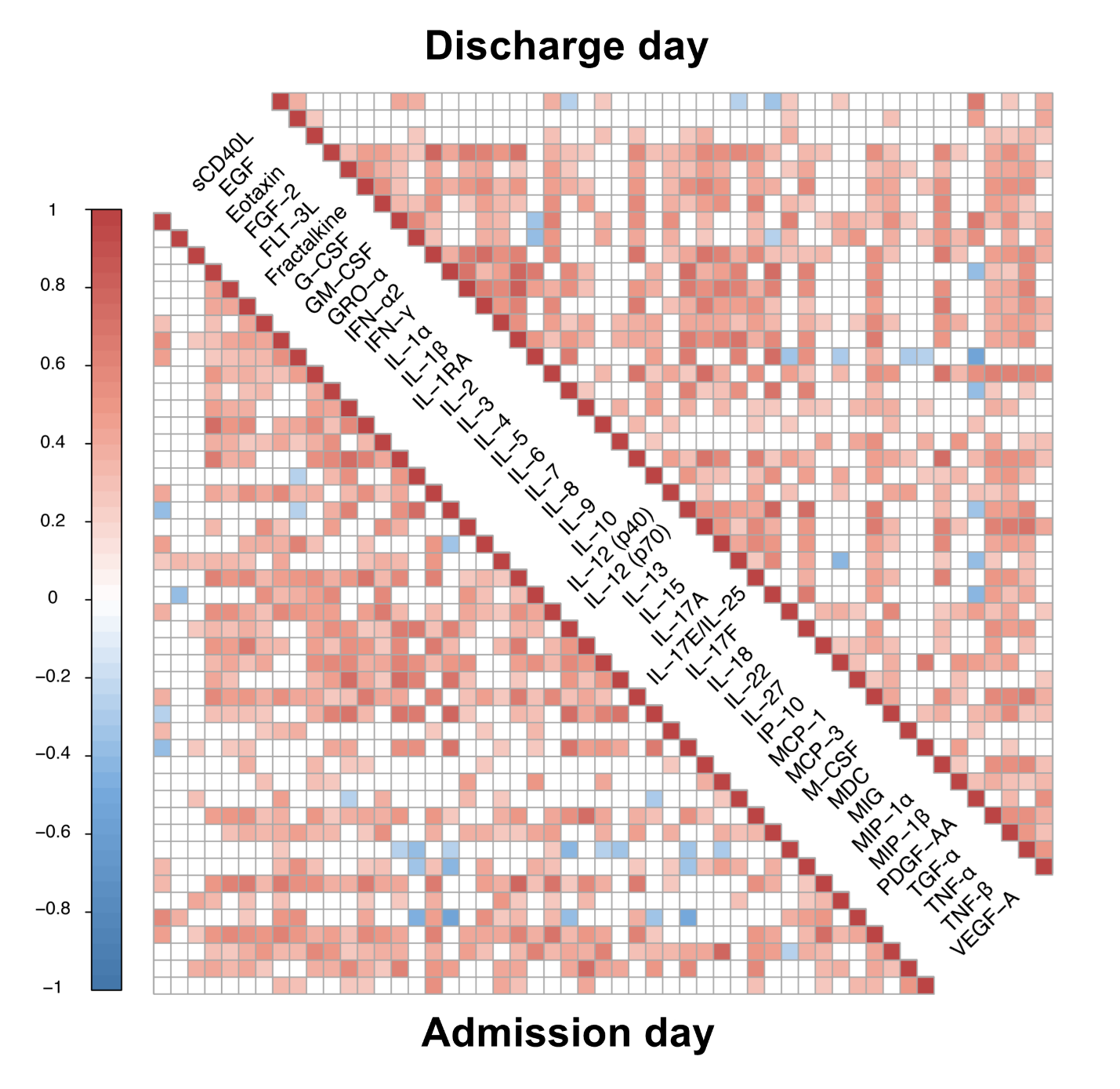
**

**Fig. S6**. **Results of correlation analysis of cytokine levels in “SCG” patients on the admission day and the discharge day.** **A.** Correlation matrices for 63 patients comparing cytokine patterns of the acute phase (the admission day) and convalescent phase (the discharge day). Colors indicate Spearman correlation coefficients (*p<*0.05), and colorless squares indicate ns (not significant) values.

**
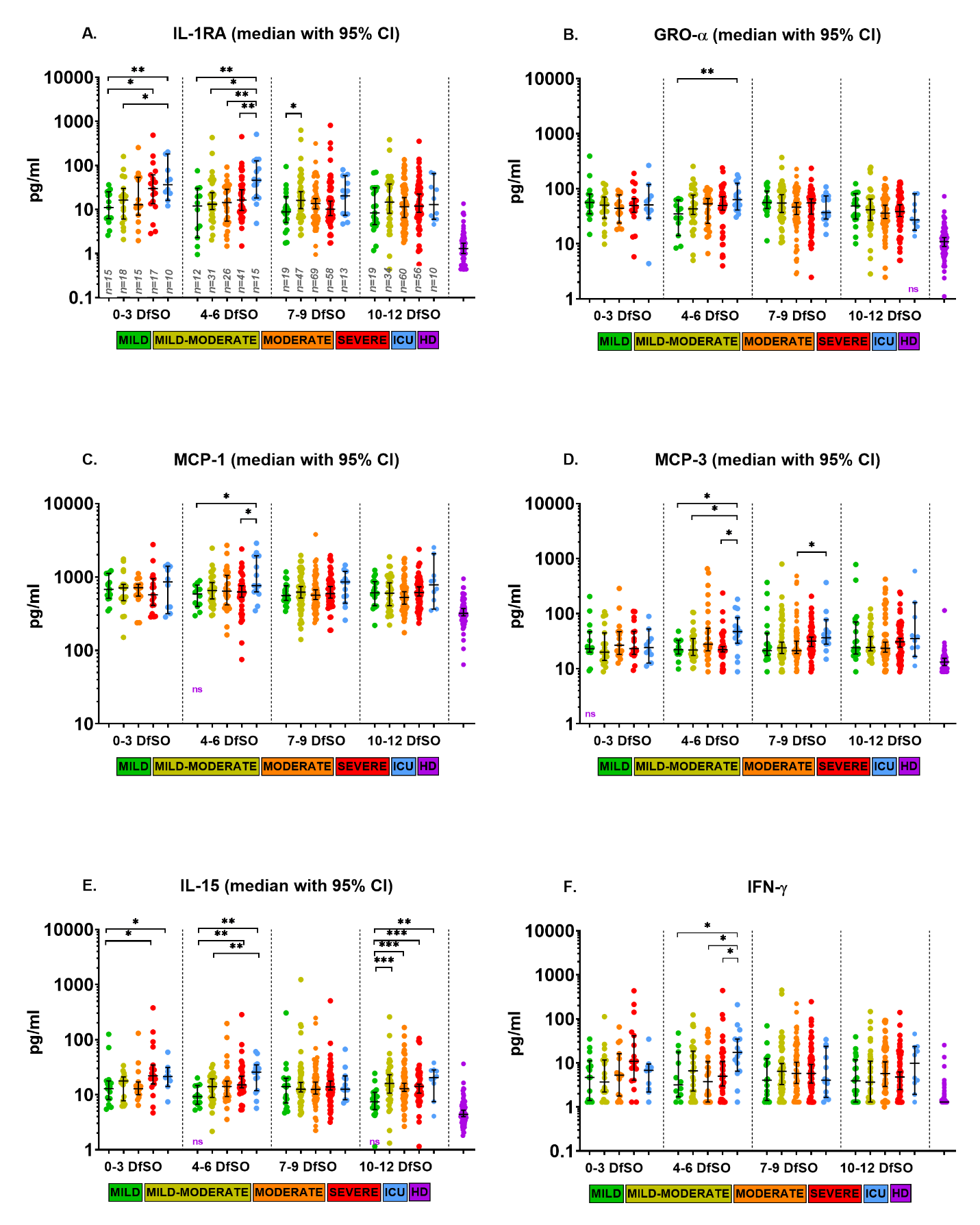
**

**Fig. S7. Dynamics of serum cytokine levels demonstrating differences between severity groups of COVID-19 patients in the first 12 days after illness onset.** **A-F** show serum levels of IL-1RA, GRO-**α**, MCP-3, MCP-1, IL-15, and IFN-g in patients with different COVID-19 severities at days 0–3, 4–6, 7-9, and 10-12 DfSO. Dots show individual measurements, and lines represent medians with 95% CIs. Statistical analyses were performed with a two-tailed Mann–Whitney U-test for nonparametric comparison. **p<*0.05*, **p<*0.01*, ***p<*0.001*, ****p<*0.0001*,* *ns -* not significant.

**Supplementary Tables**

**Table S1.** Basic demographic of cohort of 444 COVID-19 patients.

| Characteristics | Healthy donors | All patients | Disease severity | | | | |
| --- | --- | --- | --- | --- | --- | --- | --- |
|  |  |  | **Mild** | **Mild-moderate** | **Moderate** | **Severe** | **ICU** |
|  | N=66 | N=444 | N=41 | N=98 | N=137 | N=129 | N=39 |
| Percent of total | NA* | 100% | 9% | 22% | 31% | 29% | 9% |
| Median age (95% CI), years | 59,5 (30 - 74,3) | 60 (28 - 85,23) | 48 (26,1 - 84,4) | 61 (30,38 - 88,05) | 57 (20,45 - 82) | 62 (36,25 - 89,25) | 69 (24 - 93) |
| Sex, female/male, no. (%) | 58 (88)/8 (12) | 247 (56)/197 (44) | 24 (60)/16 (40) | 59 (60)/40 (40) | 74 (54)/63 (46) | 69 (53)/59 (47) | 20 (51)/19 (49) |
| Median interval from symptoms onset on admission (95% CI), days | NA | 7 (0 - 29,8) | 5 (0 - 20) | 7 (9 - 31,05) | 8 (0 - 28,75) | 7 (0 - 30,33) | 6 (0 -31) |
| Median days of hospitalization (95% CI), days | NA | 12,5 (4 - 27) | 11 (3 - 23,7) | 12 (2 - 21) | 13 (2,35 - 24,55) | 13 (5,25 - 37,75) | 13 (4 - 41) |
| SARS-CoV-2 PCR positive patients, no. (%) | NA | 298 (58) | 25 (60) | 60 (61) | 95 (69) | 87 (67) | 31 (79) |
| Oxygen therapy, no. (%) | NA | 232 (52) | 1 (2) | 33 (34) | 63 (46) | 100 (78) | 35 (90) |
| Comorbidities, no. (%) | NA | 325 (73) | 22 (54) | 73 (74) | 95 (69) | 100 (78) | 35 (90) |
| In-hospital mortality, no (%) | NA | 28 (6) | 0 (0) | 0 (0) | 0 (0) | 1 (0,8) | 27 (69) |
|  |  |  |  |  |  |  |  |
| Differentiation criteria of severity | | | | | | | |
| Chest CT findings on admission |  |  |  |  |  |  |  |
| Abnormal results, no. (%) | NA | 405 (91) | 32 (78) | 86 (88) | 125 (91) | 128 (99) | 34 (87) |
| no CT changes | NA | 26 (6) | 7 (17) | 6 (6) | 10 (7) | 0 (0) | 3 (8) |
| no information | NA | 13 (3) | 2 (5) | 6 (6) | 2 (1) | 1 (1) | 2 (5) |
| Degree of involvement, no. (%) |  |  |  |  |  |  |  |
| ≤ 50% (CT score 1-2) | NA | 267 (60) | 26 (63) | 71 (72) | 98 (72) | 56 (43) | 16 (41) |
| > 50% (CT score 3-4) | NA | 73 (16) | 0 (0) | 0 (0) | 0 (0) | 59 (46) | 14 (36) |
| score unknown | NA | 65 (15) | 6 (15) | 15 (15) | 27 (20) | 13 (10) | 4 (10) |
| Saturation of oxygen (SpO_2_), no. (%) |  |  |  |  |  |  |  |
| >93% | NA | 330 (74) | 41 (100) | 97(99) | 132 (96) | 41 (32) | 19 (49) |
| ≤93% | NA | 105 (24) | 0 (0) | 0 (0) | 0 (0) | 87 (67) | 18 (46) |
| unknown | NA | 9 (2) | 0 (0) | 1 (1) | 5 (4) | 1 (0,7) | 2 (5) |
| Respiratory rate, breaths per min (bpm), no. (%) |  |  |  |  |  |  |  |
| ≤22 bpm | NA | 174 (39) | 41 (100) | 97 (99) | 2 (1) | 25 (19) | 9 (23) |
| >22 bpm | NA | 269 (61) | 0 (0) | 1 (1) | 135 (99) | 103 (80) | 30 (70) |
| unknown | NA | 1 (0,2) | 0 (0) | 0 (0) | 0 (0) | 1 (0,7) | 0 (0) |
| Fever, no. (%) |  |  |  |  |  |  |  |
| no fever | NA | 17 (4) | 0 (0) | 6 (6) | 4 (3) | 6 (5) | 1 (0) |
| 37-38°C | NA | 167 (38) | 41 (100) | 31 (32) | 40 (29) | 45 (35) | 10 (26) |
| ≥38 °C | NA | 247 (56) | 0 (0) | 57 (58) | 92 (67) | 72 (56) | 26 (67) |
| value unknown | NA | 13 (3) | 0 (0) | 4 (4) | 1 (1) | 6 (5) | 2 (5) |
|  |  |  |  |  |  |  |  |
| Symptoms on admission, no. (%) | | | | | | | |
| Fatigue | NA | 399 (90) | 33 (80) | 81 (83) | 131 (96) | 120 (93) | 34 (87) |
| Headache | NA | 60 (14) | 5 (12) | 15 (15) | 24 (18) | 14 (11) | 2 (5) |
| Muscle pain | NA | 61 (14) | 2 (5) | 15 (15) | 27 (20) | 16 (12) | 1 (3) |
| Cough | NA | 397 (89) | 26 (63) | 93 (95) | 125 (91) | 118 (91) | 35 (90) |
| Pharyngalgia | NA | 77 (17) | 11 (27) | 19 (19) | 20 (15) | 20 (16) | 7 (18) |
| Dyspnea | NA | 291 (66) | 0 (0) | 64 (65) | 97 (71) | 98 (76) | 32 (82) |
| Nasal congestion | NA | 30 (7) | 7 (17) | 5 (5) | 12 (9) | 3 (2) | 3 (8) |
| Dizziness | NA | 37 (8) | 1 (2) | 13 (13) | 8 (6) | 13 (10) | 2 (5) |
| Loss of smell | NA | 55 (12) | 6 (15) | 11 (11) | 24 (18) | 13 (10) | 1 (3) |
| Loss of taste | NA | 28 (6) | 3 (7) | 3 (3) | 8 (6) | 14 (11) | 0 (0) |
| Stomachache | NA | 28 (6) | 4 (10) | 8 (8) | 9 (7) | 6 (5) | 1 (3) |
| Diarrhea | NA | 142 (32) | 14 (34) | 35 (36) | 53 (39) | 34 (26) | 6 (15) |
| Nausea/vomiting | NA | 76 (17) | 7 (17) | 17 (17) | 29 (21) | 21 (16) | 2 (5) |
|  |  |  |  |  |  |  |  |

*NA – not applicable

**Table S2.** Cytokine profiles of patients with COVID-19 in association with different noninfectious comorbidities

| **Chronic noninfectious diseases** | **Cytokines increased in COVID-19 patients with comorbidities CM+ (median, p-value)** | **Cytokines lowered in COVID-19 patients with comorbidities CM+ (median, p-value)** | **Possible reasons for differences of cytokine levels between COVID-19 patients with (CM+) or without (CM-) comorbidities** |
| --- | --- | --- | --- |
| **Cardiovascular diseases (CVD)** | IL-1RA (33.5, *p*<0.01) | EGF (376, *p*<0.01) | Imbalance between IL-1 and **IL-1RA** can lead to coronary heart disease. To inhibit pathological functions of IL-1, more than 100 times higher amount of IL-1RA is needed(11). |
|  | IL-6 (24, *p*<0.05) | IL-13 (44, *p*<0.05) | Levels of **IL-6** and IL-10 are increased in patients with CVD, especially in those with hypertension and coronary heart disease(12). |
| **Chronic obstructive pulmonary disease (COPD)** | IL-1RA (36, *p*<0.05) | sCD40L  (7801, *p<0.05*) | Imbalance between IL-1/**IL-1RA** can lead to the development of lung diseases in animal models and in humans, such as fibrotic and infectious lung diseases, particularly granuloma formation. Endogenous IL-1Ra was shown to exert important anti-inflammatory effects in a model of immune lung injury, as produced by both macrophages and infiltrating neutrophils. A high concentration in bronchoalveolar lavage correlates with a good outcome in, for example, adult respiratory distress syndrome(11). |
|  |  | IL-13 (44, *p*<0.05) |  |
|  | IL-6 (33, *p*<0.05) | IL-17A (9, *p*<0.05) | In smokers with COPD progression, the level of serum **IL-6** is increased(13). |
|  | MCP-1 (765, *p*<0.05) | IL-22 (16, *p*<0.05) | The blood level of **MCP-1** is increased in patients with COPD with the progression of emphysema(14). |
|  | MIG (3744, *p*<0.05) | TNF-β (18.5, *p*<0.05) | In patients with COPD and emphysema, secretion of **MIG**, IP-10, IFN-γ by CD4/CD8 T-helpers with Th1 phenotype is increased(15). |
| **Bronchial asthma (BA) +COPD** | IL-6 (31, *p*<0.05) | sCD40L  (7801, *p*<0.01) | IL-5, **IL-6**, IL-13 and IL-17A are increased in patients with BA(16). |
|  |  | EGF (376, *p*<0.05) |  |
|  |  | IL-13 (44, *p*<0.05) |  |
| **Diabetes mellitus (DM)** | IL-6 (25, *p*<0.05) | IL-1β (17, *p*<0.05) | **IL-6**, IL-2, IL-10 and INF-γ are upregulated in patients with DM compared to healthy controls.  **IL-6**, IL-8, and TNF-α function as pro-inflammatory cytokines and induce insulin-resistance development and β-cells dysfunction, leading to the progression of type II DM.  IL-6 regulates expression of IL-10 and INF-γ and inhibits that of TNF-α and **IL-1β** during DM(17). |
|  | IL-1RA (35, *p*<0.05) |  | **IL-1RA** inhibits IL-1-induced decreases in insulin secretion by pancreatic cells in vitro. It was hypothesized that an imbalance between IL-1 and IL-1RA may predispose toward the development of DM and that high levels of IL-1RA are required to inhibit the injurious effects of IL-1 on cell function(11). |
|  |  | EGF (376, *p*<0.01) | In diabetes mice models, EGF secretion in the submandibular gland and its concentration in plasma are reduced compared to in healthy controls, at 13% and 30%, respectively(18). |
|  |  | IL-13 (44, *p*<0.05) | **IL-13** is increased in the blood of diabetics with retinopathy(19). |
| **Rheumatic disease (rheumatoid arthritis+ Hashimoto's thyroiditis)** | MCP-1 (837, *p*<0.05) | EGF (376, *p*<0.05) | **MCP-1** in plasma is an indicator of inflammation during RA(20). |
|  | MIG (4768, *p*<0.01) | TNF-β (18.5, *p*<0.05) | Levels of **MIG**, IP10, PF4 in synovial fluid and tissues in patients with RA are increased and can suppress inflammation and reduce angiogenesis(21).  Levels of **MIG** and its receptor CXCR3 are increased during the development of autoimmune thyroiditis(22). |
| **Obesity** | IL-6 (33, *p*<0.05) | EGF (376, *p*<0.05) | Main pro-inflammatory cytokines of adipose tissue - **TNF-α**, **IL-6**, and IL-1(23). |
|  | TNF-α (119, *p*<0.05) |  |  |
|  | IL-12 (p40) (90, *p*<0.05) |  | During obesity, under a high-fat diet (HFD), pro-inflammatory TH1-cells and M1 macrophages are activated and secretion of IFN-γ, **TNF-α**, and **IL-12** is increased; secretion of IL-4, IL-10 and IL-13 (Th2) is reduced.  Gene expression of IL-12 and other IL-12 family members is upregulated in insulin-responsive tissue, such as the skeletal muscle, liver, and heart, due to excessive nutrient intake, inflammatory stress and genetic obesity(24). |
|  | IP-10 (1006, *p*<0.05) |  | **IP-10(CXCL10)** and CXCL11 are potential inhibitors of angiogenesis of adipose tissue during obesity progression(25). |
|  | MCP-1 (744, *p*<0.05) |  | **MCP-1** has significant effects in nonalcoholic fatty liver disease (NAFLD), obesity, and other lipid overload states. Its plasma level is increased in patients with obesity independently of their age(26). |
|  |  |  | Hyperplastic or hypertrophied adipose tissues directly secrete various inflammatory products, such as inflammatory cytokines, TGF-β, adipokines, **MCP-1**, CXCL 5, hemostatic proteins, proteins affecting blood pressure, and angiogenic molecules(23). |
|  | MIG (4105, *p*<0.01) |  | **MIG** – no direct connection, though its receptor may be involved in immunological processes in I type diabetes(26). |

**Table S3. Questionnaire for COVID-19 patients from Clinic of Infection Disease №1, Moscow, Russia**

**Questionnaire COVID-19**

***Clinic of Infection Disease №1 of Moscow Healthcare Department, 2020***

1. **Hospital admission** (dd/mm/yyyy) |__|__| / |__|__| / |__|__|__|__|
2. **Hospital department №** |__|__|
3. **Case history № (Patient`s identical number):** __________________
4. **Sex** |__| Male |__| Female
5. **Age**_________________ **Date of birth** (dd/mm/yyyy) |__|__| / |__|__| / |__|__|__|__|
6. **Patient had contact with a possible or confirmed case of COVID-19**

|__| Yes |__| No

1. **Patient had close contact with a patient with ARI(acute respiratory infections) within 14 days before the symptoms onset** |__| Yes |__| No
2. **Patient was abroad within 14 days before symptoms onset**

|__| Yes* |__| No

*if the answer was “Yes”, fill in points 9-11.

1. **Dates of travel** (dd/mm/yyyy) **from** |__|__| / |__|__| / |__|__|__|__| till |__|__| / |__|__| / |__|__|__|__|
2. **Country** _______________________________________________
3. **Patient was PCR SARS-CoV-2 tested at the border control** |__| Yes |__| No
4. **Date of self-isolation** (dd/mm/yyyy) |__|__| / |__|__| / |__|__|__|__|
5. **Day from symptoms onset** (dd/mm/yyyy) |__|__| / |__|__| / |__|__|__|__|

**а) Main symptoms**

a. Fever |__| 37-38º |__| 38-39º |__| 39-40º |__|

b. Fatigue |__|

c. Headache |__|

d. Muscle pain |__|

e. Rash |__|

**b) Respiratory symptoms**

a. Cough |__|

b. Sore throat |__|

c. Dyspnea |__|

d. Nasal congestion |__|

**c) Neurological symptoms**

a. Impaired consciousness |__|

b. Meningeal/focal signs |__|

c. Dizziness |__|

d. Loss of smell |__|

e. Loss of taste |__|

**d) Gastrointestinal symptoms**

a. Stomach |__|

b. Diarrhea |__|

c. Nausea/vomiting |__|

**14.** **Chest CT findings on admission** |__| Yes |__| No Degree of involvement (CT score) ____________________

**NASOPHARYNGEAL SWAB SAMPLES**

**15. Date of nasopharyngeal swab sample** (dd/mm/yyyy) admission day |__|__| / |__|__| / |__|__|__|__|

**16. Date of nasopharyngeal swab sample** (dd/mm/yyyy) |__|__| / |__|__| / |__|__|__|__|

**17. Date of nasopharyngeal swab sample** (dd/mm/yyyy) discharge day |__|__| / |__|__| / |__|__|__|__|

**BLOOD SAMPLES**

**18. Date of blood sample** (dd/mm/yyyy) admission day |__|__| / |__|__| / |__|__|__|__|

**19. Date of blood sample** (dd/mm/yyyy) |__|__| / |__|__| / |__|__|__|__|

**20. Date of blood sample** (dd/mm/yyyy) discharge day |__|__| / |__|__| / |__|__|__|__|

**PATIENT`S CHARACTERISTICS**

**21. Chronic diseases:**

| a. Cardio-vascular diseases | \|__\| |
| --- | --- |
| b. Chronic obstructive pulmonary disease | \|__\| |
| c. Bronchial asthma | \|__\| |
| d. Diabetes mellitus | \|__\| |
| e. Immunodeficiency (except HIV) | \|__\| |
| f. Kidney disease | \|__\| |
| g. Rheumatological/Autoimmune diseases | \|__\| |
| h. Neurological diseases | \|__\| |
| i. Cirrhosis/liver disease | \|__\| |
| j. Cancer  k. Obesity  l. Tuberculosis (active form)  m. HIV  p. Other | \|__\|  \|__\|  \|__\|  \|__\|  \|__\| |

**22. Antiviral therapy before hospitalization (during symptoms onset)**

|__| Yes |__| No |__| Unknown Which drugs? ____________________________________________

**23. Antibiotics therapy before hospitalization (during symptoms onset)**

|__| Yes |__| No |__| Unknown Which drugs? ____________________________________________

**24. Antiviral therapy during hospitalization**

|__| Yes |__| No |__| Unknown Which drugs? ____________________________________________

**25. Antibiotics therapy during hospitalization**

|__| Yes |__| No |__| Unknown Which drugs? ____________________________________________

**26. Immunosuppressive therapy before hospitalization (during symptoms onset)**

|__| Yes |__| No |__| Unknown Which drugs? ____________________________________________

**27. Immunosuppressive therapy during hospitalization (Tocilizumab)**

|__| Yes |__| No |__| Unknown Which drugs? ____________________________________________

**CRITERIA OF SEVERITY**

**28. Disorientation upon admission** |__| Yes |__| No

**29. Blood pressure** |__|__|__|__| /__|__|__|__mmHg

**30. Respiratory rate** (breaths per min, bpm) |__|__|

**31.** **Oxygen support without mechanical ventilation** |__| Yes |__| No

**32. Vasopressor support** |__| Yes |__| No

**33. Oxygen support with mechanical ventilation** |__| Yes |__| No

**34. Apnea** |__| Yes |__| No

**DISEASE OUTCOME**

**35. ICU** |__| Yes |__| No

**36. Death during hospitalization** |__| Yes |__| No

**37. Date of death/discharge** (dd/mm/yyyy) |__|__| / |__|__| / |__|__|__|__|

**38. Transfer to another hospital** |__| Yes |__| No

**39. a. Main 1 diagnosis (ICD-10)**

|__| / |__|__|__| . |__|__|

**b. Secondary 2 diagnosis (Complications)**

|__| / |__|__|__| . |__|__| Pneumonias |__| Yes |__| No

Bronchitis |__| Yes |__| No

**c. Secondary 3 diagnosis (Chronic diseases)**

|__| / |__|__|__| . |__|__|

**RESULTS OF PCR**

| **Result** | **Admission day** | **5 ± 2 day** | **Discharge day** |
| --- | --- | --- | --- |
| **Positive COVID-19** | \|__\|Yes\|__\|No | \|__\|Yes\|__\|No | \|__\|Yes\|__\|No |

**Another respiratory virus infection** |__|Yes* |__|No

*if the answer was “Yes”, point the infection__________________________________________________________

**Supplementary references**

1. Long Q-X, Liu B-Z, Deng H-J, Wu G-C, Deng K, Chen Y-K, et al. Antibody responses to SARS-CoV-2 in patients with COVID-19. Nat Med. 2020 Jun;26(6):845–8.

2. Zhao J, Yuan Q, Wang H, Liu W, Liao X, Su Y, et al. Antibody Responses to SARS-CoV-2 in Patients With Novel Coronavirus Disease 2019. Clin Infect Dis. 2020 Nov 19;71(16):2027–34.

3. Lou B, Li T-D, Zheng S-F, Su Y-Y, Li Z-Y, Liu W, et al. Serology characteristics of SARS-CoV-2 infection since exposure and post symptom onset. Eur Respir J [Internet]. 2020 May 19 [cited 2021 Apr 21]; Available from: https://www.ncbi.nlm.nih.gov/pmc/articles/PMC7401320/

4. COVID-19 Clinical management: living guidance [Internet]. 2020 [cited 2021 Apr 20]. Available from: https://www.who.int/publications/i/item/WHO-2019-nCoV-clinical-2021-1

5. Del Valle DM, Kim-Schulze S, Huang H-H, Beckmann ND, Nirenberg S, Wang B, et al. An inflammatory cytokine signature predicts COVID-19 severity and survival. Nat Med. 2020 Oct;26(10):1636–43.

6. Satış H, Özger HS, Aysert Yıldız P, Hızel K, Gulbahar Ö, Erbaş G, et al. Prognostic value of interleukin-18 and its association with other inflammatory markers and disease severity in COVID-19. Cytokine. 2021 Jan;137:155302.

7. Petrey AC, Qeadan F, Middleton EA, Pinchuk IV, Campbell RA, Beswick EJ. Cytokine release syndrome in COVID-19: Innate immune, vascular, and platelet pathogenic factors differ in severity of disease and sex. J Leukoc Biol. 2021;109(1):55–66.

8. Klein SL, Flanagan KL. Sex differences in immune responses. Nat Rev Immunol. 2016 Oct;16(10):626–38.

9. Capuano A, Rossi F, Paolisso G. Covid-19 Kills More Men Than Women: An Overview of Possible Reasons. Front Cardiovasc Med [Internet]. 2020 [cited 2021 Apr 21];7. Available from: https://www.frontiersin.org/articles/10.3389/fcvm.2020.00131/full

10. Angioni R, Sánchez-Rodríguez R, Munari F, Bertoldi N, Arcidiacono D, Cavinato S, et al. Age-severity matched cytokine profiling reveals specific signatures in Covid-19 patients. Cell Death Dis. 2020 Nov 6;11(11):1–12.

11. Arend WP. The balance between IL-1 and IL-1Ra in disease. Cytokine Growth Factor Rev. 2002 Aug 1;13(4):323–40.

12. Li M, Dong Y, Wang H, Guo W, Zhou H, Zhang Z, et al. Cardiovascular disease potentially contributes to the progression and poor prognosis of COVID-19. Nutr Metab Cardiovasc Dis. 2020 Jun 25;30(7):1061–7.

13. Emami Ardestani M, Zaerin O. Role of Serum Interleukin 6, Albumin and C-Reactive Protein in COPD Patients. Tanaffos. 2015;14(2):134–40.

14. Stefano AD, Coccini T, Roda E, Signorini C, Balbi B, Brunetti G, et al. Blood MCP-1 levels are increased in chronic obstructive pulmonary disease patients with prevalent emphysema. Int J Chron Obstruct Pulmon Dis. 2018 May 24;13:1691–700.

15. Grumelli S, Corry DB, Song L-Z, Song L, Green L, Huh J, et al. An Immune Basis for Lung Parenchymal Destruction in Chronic Obstructive Pulmonary Disease and Emphysema. PLOS Med. 2004 Oct 19;1(1):e8.

16. Dimitrova D, Youroukova V, Ivanova-Todorova E, Tumangelova-Yuzeir K, Velikova T. Serum levels of IL-5, IL-6, IL-8, IL-13 and IL-17A in pre-defined groups of adult patients with moderate and severe bronchial asthma. Respir Med. 2019 Jul 1;154:144–54.

17. Zheng M, Wang X, Guo H, Fan Y, Song Z, Lu Z, et al. The Cytokine Profiles and Immune Response Are Increased in COVID-19 Patients with Type 2 Diabetes Mellitus. J Diabetes Res. 2021 Jan 2;2021:e9526701.

18. Kasayama S, Ohba Y, Oka T. Epidermal growth factor deficiency associated with diabetes mellitus. Proc Natl Acad Sci. 1989 Oct 1;86(19):7644–8.

19. Hang H, Yuan S, Yang Q, Yuan D, Liu Q. Multiplex bead array assay of plasma cytokines in type 2 diabetes mellitus with diabetic retinopathy. Mol Vis. 2014 Aug 4;20:1137–45.

20. Ellingsen T, Buus A, Stengaard-Pedersen K. Plasma monocyte chemoattractant protein 1 is a marker for joint inflammation in rheumatoid arthritis. J Rheumatol. 2001 Jan 1;28(1):41–6.

21. Zoltan S, Gabriella S, Sandor S, Alisa EK. Chemokines in Rheumatic Diseases. Curr Drug Targets. 2005 Dec 31;7(1):91–102.

22. Pezzano M. MIG in autoimmune thyroiditis: review of the literature. Clin Ter. 2019 Aug;170(4):e295–300.

23. Lim S, Shin SM, Nam GE, Jung CH, Koo BK. Proper Management of People with Obesity during the COVID-19 Pandemic. J Obes Metab Syndr. 2020 Jun 30;29(2):84–98.

24. Schmidt FM, Weschenfelder J, Sander C, Minkwitz J, Thormann J, Chittka T, et al. Inflammatory Cytokines in General and Central Obesity and Modulating Effects of Physical Activity. PLOS ONE. 2015 Mar 17;10(3):e0121971.

25. Hueso L, Ortega R, Selles F, Wu-Xiong NY, Ortega J, Civera M, et al. Upregulation of angiostatic chemokines IP-10/CXCL10 and I-TAC/CXCL11 in human obesity and their implication for adipose tissue angiogenesis. Int J Obes. 2018 Aug;42(8):1406–17.

26. Xue W, Fan Z, Li L, Lu J, Zhai Y, Zhao J. The chemokine system and its role in obesity. J Cell Physiol. 2019;234(4):3336–46.
